# Occupancy and detectability modelling of vertebrates in northern Australia using multiple sampling methods

**DOI:** 10.1371/journal.pone.0203304

**Published:** 2018-09-24

**Authors:** Luke D. Einoder, Darren M. Southwell, José J. Lahoz-Monfort, Graeme R. Gillespie, Alaric Fisher, Brendan A. Wintle

**Affiliations:** 1 Flora and Fauna Division, Department of Environment and Natural Resources, Darwin, Northern Territory, Australia; 2 Quantitive and Applied Ecology Group, School of BioSciences, University of Melbourne, Melbourne, Victoria, Australia; University of Sydney, AUSTRALIA

## Abstract

Understanding where species occur and how difficult they are to detect during surveys is crucial for designing and evaluating monitoring programs, and has broader applications for conservation planning and management. In this study, we modelled occupancy and the effectiveness of six sampling methods at detecting vertebrates across the Top End of northern Australia. We fitted occupancy-detection models to 136 species (83 birds, 33 reptiles, 20 mammals) of 242 recorded during surveys of 333 sites in eight conservation reserves between 2011 and 2016. For modelled species, mean occupancy was highly variable: birds and reptiles ranged from 0.01–0.81 and 0.01–0.49, respectively, whereas mammal occupancy was lower, ranging from 0.02–0.30. Of the 11 environmental covariates considered as potential predictors of occupancy, topographic ruggedness, elevation, maximum temperature, and fire frequency were retained more readily in the top models. Using these models, we predicted species occupancy across the Top End of northern Australia (293,017 km^2^) and generated species richness maps for each species group. For mammals and reptiles, high richness was associated with rugged terrain, while bird richness was highest in coastal lowland woodlands. On average, detectability of diurnal birds was higher per day of surveys (0.33 ± 0.09) compared with nocturnal birds per night of spotlighting (0.13 ± 0.06). Detectability of reptiles was similar per day/night of pit trapping (0.30 ± 0.09) as per night of spotlighting (0.29 ± 0.11). On average, mammals were highly detectable using motion-sensor cameras for a week (0.36 ± 0.06), with exception of smaller-bodied species. One night of Elliott trapping (0.20 ± 0.06) and spotlighting (0.19 ± 0.06) was more effective at detecting mammals than cage (0.08 ± 0.03) and pit trapping (0.05 ± 0.04). Our estimates of species occupancy and detectability will help inform decisions about how best to redesign a long-running vertebrate monitoring program in the Top End of northern Australia.

## Introduction

Monitoring is important for determining the status and trends of species as well as their response to threats or management intervention. Despite the importance of monitoring, most programs are under-resourced [1], placing constraints on their size (number of sites), scope (single or multiple species), spatial scale, sampling intensity, and the number and types of sampling methods. These constraints increase the need for well-conceived, designed and implemented monitoring programs. Ideally, the number and location of monitoring sites should overlap with the distribution of focal species at an appropriate spatial scale to draw accurate inference about trends in the broader population [2]. The sampling methods and intensity should also ensure a high chance of detecting species that are present at sites, and ensure monitoring has adequate statistical power to detect changes in abundance/distributions over space and time [3,4]. Occupancy and detectability modelling can inform such decisions, and is growing in popularity as practitioners strive for more cost-effective biodiversity monitoring [5,6].

In northern Australia, long-term monitoring has been crucial for documenting the status and trends of vertebrate communities, especially a drastic decline in small-to-medium sized mammals from 1996–2009 [7–9]. Much of the evidence for these trends arises from monitoring programs in large and relatively well-resourced conservation reserves [7,8,10]. The most informative and substantial of these programs has been the ‘Three Parks Program’ in Kakadu, Litchfield and Nitmiluk National Parks, which was established in 1996 to track the status and population trends of faunal diversity [11]. Over the last 20 years, presence-absence and abundance data has been collected at approximately 250 sites within these parks at five to six-yearly intervals for 398 species of native bird, mammal, reptile and amphibian (http://nrmaps.nt.gov.au/nrmaps.html). These monitoring data have been instrumental for setting conservation priorities in the region [8], elucidating the role of potential drivers of species abundance and distributions [12], and for informing management practices.

In response to these declines, the agency responsible for the Three Parks Program, Department of Environment and Natural Resources of the Northern Territory Government, has sought to redesign the monitoring program. One motivation for a redesign is that the initial location of sites and frequency of sampling was not chosen with vertebrate species in mind: rather, it was super-imposed over an existing monitoring program design to learn about the effect of fire on vegetation [11]. A lack of spatial coverage of sites across the Top End has also constrained knowledge about the status of species at a broader scale [13], and might limit identification of potential drivers of species occupancy across the landscape. The primary objectives of a redesigned program are to: improve the statistical power of monitoring to detect trends in species should future changes in populations occur; and, improve the ability to draw inference about faunal diversity in un-sampled areas. An important step in the redesign is to consider the location and spatial arrangement of sites with respect to current levels of species occupancy/richness in the landscape.

An additional motivation for redesigning the Three Parks Program is to identify and use only the most effective methods in a way that maximises the chance of detecting species, should they occur at sites. Many species are detected at very low frequencies; however, it is not clear whether this is due to low levels of occupancy, or because species are present but not detected during surveys. Few survey methods detect all individual animals, or even all species of animals, in surveyed areas. Quantifying detectability for focal species with existing data will inform which sampling methods are most efficient, as well as how much monitoring effort is needed to confidently detect species that are present [14,15]. Studies comparing the effectiveness of vertebrate sampling methods do exist (e.g. camera trapping [16,17]), but are surprisingly rare [17, 18]. Estimates of both occupancy and detectability are required to calculate the statistic power of monitoring to detect changes in occupancy, or the level of sampling effort needed to detect a desired level of change with sufficient power [5,6].

As a first step in the redesign of the Three Parks Program, we used existing repeat detection/non-detection data collected from 333 sites in eight conservation reserves to: 1) estimate occupancy of birds, mammals and reptiles at monitoring sites, as well as across the broader Top End of northern Australia; 2) estimate the effectiveness of six sampling methods (live trapping methods, camera trapping and active searches), and; 3) predict relative species richness across the Top End of northern Australia. Given the objective of the redesigned monitoring program will be to detect changes in all birds, mammals (excluding bats) and reptiles–not just those that are rare or threatened [19]–we attempted to fit models to all species detected at least once during the most recent round of monitoring (2011–2016). Our estimates of occupancy and detectability, combined with species richness maps will provide a foundation for assessing: the relative performance of alternative sampling methods; the spatial coverage of sites relative to where species that can be readily detected with the methods might occur; and, the chance that future monitoring will confidently detect population change.

## Materials and method

### Study area

**Fig 1:**
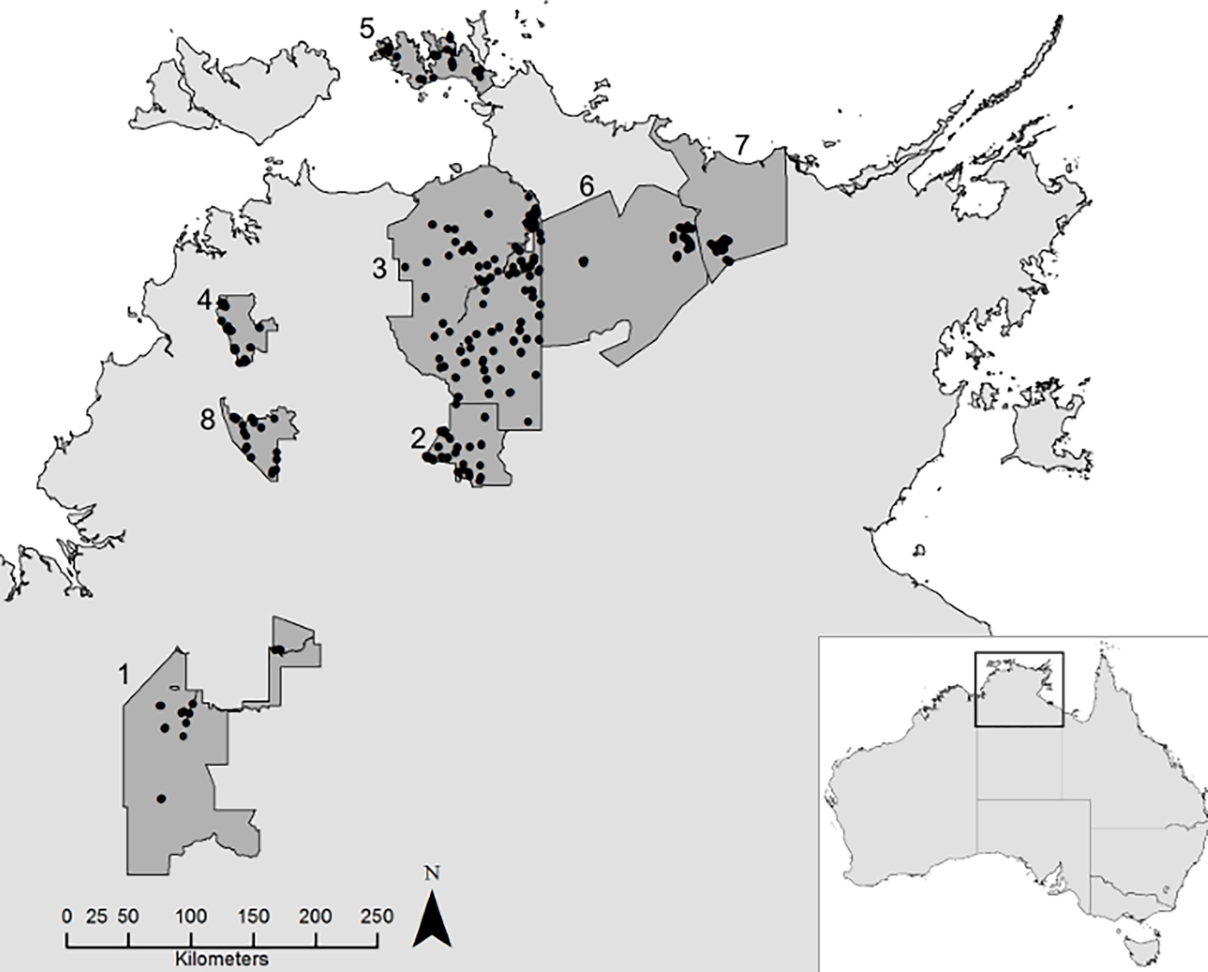
Site locations sampled between 2011 and 2016 using multiple methods. Location of 333 monitoring sites (black dots) in eight protected areas (1-Gregory National Park, 2-Nitmiluk National Park, 3-Kakadu National Park, 4-Litchfield National Park, 5-Garig Gunak Barlu National Park, 6-Warddeken IPA, 7-Djelk IPA, 8-Fish River Station) in the Top End of the Northern Territory, Australia, from which presence-absence data for mammals, reptiles and birds was collected.

Our study area was the northern section of the Northern Territory of Australia, commonly known as the ‘Top End’ (Fig 1). The region is subject to a wet-dry tropical climate where annual rainfall ranges between ~1400 mm in the north to 500 mm in the south, of which about 90% falls during a pronounced wet season (typically November-May). Tropical savanna extends as far south as the 500 mm annual rainfall isohyet [20], and contains large areas of eucalypt woodlands, eucalypt open forest, and smaller areas of monsoon rainforest, Allosyncarpia forest, sandstone heath, floodplain, wetland and riparian communities [21].

### Data collation

We utilised presence-absence data from the most recent round of a long-running vertebrate monitoring program within Kakadu, Litchfield and Nitmiluk national parks [11], combined with data from surveys in other areas across the study area. In total, we collated repeat detection/non-detection data from 333 sites across five national parks (Kakadu, Litchfield, Nitmiluk, Garig Gunak Barlu, and Gregory), two Indigenous Protected Areas (IPA; Warddeken and Djelk), and one privately owned conservation reserve (Fish River Station) (Fig 1). Sites within each reserve stratify all major vegetation types within dryland habitats of the Top End. We limited our dataset to surveys conducted between 2011 and 2016 to provide an up-to-date snapshot of species occupancy.

All sites were visited once and surveyed for mammals, birds and reptiles using a standard protocol [8], although the timing of visits to sites within a year varied between parks. Sites consisted of a 50 x 50 m quadrat (hereafter referred to as the trapping quadrat) containing: 16 Elliott traps placed equidistantly around the perimeter; eight cage traps placed at the corners and mid-way along each side of the quadrat; and three 20-L pitfall traps with 10 m of drift fence. Cage and Elliott traps were baited with a mixture of peanut butter, honey and oats, set at dusk and checked and closed at dawn. Pitfall traps were checked for reptiles and mammals at dawn, midday and dusk. Cage, Elliott and pit trapping was conducted for three or four days/nights at each site. Surveys were completed under Charles Darwin University Animal Ethics Permit A13026, and all sampling procedures were specifically approved as part of obtaining the field permit.

At a subset of sites, diurnal bird surveys (225 sites), nocturnal searches (228 sites), and camera trapping (163 sites) were conducted across a 100 x 100 m quadrat centred on the trapping quadrat (S1 Table). Three ten-minute bird surveys were conducted per day over three days. Additionally, one fifteen-minute nocturnal search by spotlight was conducted on each of three nights after dark targeting reptiles, mammals and birds, without using call-playback methods to increase detection rates. Five Reconyx® motion-sensor cameras were deployed during the four-day survey following the methods of Gillespie et al. [22] and remained operational for five weeks to detect mammals. Camera images were processed by a minimum of two experienced personnel. We acknowledge these sampling methods are inadequate for amphibians and bats. Due to the expectation of infrequent records for these poorly sampled animal groups we excluded them from the analysis.

### Detection histories

We generated detection histories for species observed at least once during the study: a 1 represented the detection of a species, a 0 represented a non-detection. We collapsed detection histories so that the sampling occasion was one day/night for live trapping, bird surveys and nocturnal searches. This resulted in three to four repeat sampling occasions to sites for these methods. For camera trapping, we collapsed detection histories so that the sampling occasion was one week, resulting in five repeat visits to sites with this method. Separate detection histories were generated for each sampling method. For example, a detection history of [0,1,0,0,1] with camera trapping and [1,0,0,0] with cage trapping meant a species was detected (one or more individuals) in the second and fifth week with cameras, and only in the first day/night with cages.

### Environmental predictor variables

We compiled a list of covariates that were: 1) thought to broadly influence occupancy and detectability of birds, mammals and reptiles across the study region; and, 2) available at the broad spatial scale of the study. These included climatic variables (mean annual rainfall, maximum temperature in the warmest quarter, minimum temperature in the coldest quarter), terrain variables (elevation, terrain ruggedness, distance to perennial creeks), environmental variables (soil clay content, vegetation cover), and fire variables. Fire variables included point-based metrics (number of fires, time since fire) and neighbourhood-based metrics (fire extent, proportion burnt and fire patchiness) to capture the spatial homogeneity of fires across space and time [12]. We acknowledge that other covariates such as predator density or habitat structure may also drive occupancy of mammals, reptiles and birds. However, such factors could not be included in our modelling because where data exists it is patchy, hence could not be mapped for the study region. We obtained raster layers of the covariates and re-sampled each one in ArcGIS software at 100 m resolution (S1 Fig) before extracting their value at sampling sites. For a detailed description of the covariates and justification for their selection see Table 1.

**Table 1:** Summary of the 13 climatic, topographic and fire covariates considered in occupancy and detectability modelling.

Covariate	Units	Range	Description and source
Maximum temp. in warmest quarter	degrees	32.4–39.2	Obtained from the BioClim database at 1 km resolution (http://www.worldclim.org/bioclim). Rainfall and temperature influence mammal, bird and reptile distributions [23].
Mean annual precipitation	mm	650–1929	
Minimum temp. in coldest quarter	degrees	9.3–20.3	
Elevation	metres	0–557	From a 90 m resolution digital elevation model. Although elevation itself has no direct influence on species survival, it is thought to be a good proxy for species that are restricted to the escarpments regions.
Terrain ruggedness		9.7x10^-9^–136	Variability (Standard Deviation) in elevation within a 3.2 km radius of each cell. Terrain roughness has been shown to influence mammal distributions [24].
Soil	Percent	0–83	A 0–30 cm depth clay content percentage raster layer was obtained from CSIRO, Australia (http://www.asris.csiro.au/) at 250 m resolution. Soil type has been reported to influence reptile distributions across the Top End of Australia [25].
Distance to watercourse	metres	0–129772	Euclidean distance of each cell to the nearest perennial creek or river. Distance to water is reported to influence mammal distributions [26].
Vegetation fractional cover	Percent	0–100	Mean proportion of each cell covered by photosynthetic vegetation [27]. Vegetation fractional cover imagery was obtained for the study region from 2000–2014 at 500 m resolution (http://www.auscover.org.au/). We used imagery from September, when annual grasses have died off and greenness is largely attributable to foliage, and calculated mean fractional cover across the 15 year period. The influence of vegetation cover has been explored for reptiles [28].
Time since fire	years	0–10	We used fire scars derived from MODIS imagery at 250 m resolution (http://modis.gsfc.nasa.gov/). MODIS data were obtained from the North Australia Fire Information (NAFI) website (http://www.firenorth.org.au/nafi2/) for the years 2000–2014. Point-based fire variables were expected to influence species detectability because fire can represent changes in vegetation structure. We expected a recently burnt plots to have higher detectability.
Fire Frequency	count	0–10	
Proportion burnt	proportion	0–1	Annual average proportion of area burnt in the surrounding 3.2 km of each site during the 5 years prior to surveying [12]
Fire extent	metres	0–5728	Mean distance of each pixel within 1.6 km of a site to unburnt vegetation, averaged across the 5 years prior to surveying [12]. This is a measure of the spatial homogeneity of fires; large values indicate that the surrounding area was dominated by repeated extensive, spatially homogenous fires.
Fire patchiness	Log (metres)	2.386–5.244	Average of the natural logarithm of distances from each pixel within 1.6 km of a site to the closest burnt-unburnt boundary, averaged across 5 years prior to surveying [12]. Large values of the patchiness index indicate the surrounding area was dominated by large, spatially homogenous patches of either burnt or unburnt vegetation.

### Data exploration

We tested site covariates for collinearity (S2 Table) and discarded one of a pair if the Spearman correlation coefficient was greater than 0.7 [29]. As a result, we discarded minimum temperature in the coldest period (correlated with rainfall) and proportion burnt (correlated with fire frequency) from any further analysis.

### Occupancy and detectability modelling

We modelled occupancy and detectability for each species assuming the result from any given survey was the outcome of two binomial processes acting simultaneously [30, 31]: 1) the probability a species was present at a site (*ψ*) over long time periods; and, 2) the probability a species was present within the site and observed in any given survey visit (*ρ*). The first binomial process, occupancy, was considered a Bernoulli random variable, such that:

$$z_{i}∼Bernoulli\left(\psi_{i}\right)$$ (1)

where *ψ* is the probability of species occupancy at site *i* and *z*_*i*_ denotes the true state of occurrence. We then assumed the observation of a species at site *i* is the outcome of another Bernoulli random variable, with the product of *z*_*i*_ and detection probability *ρ*_*ij*_ the success probability. The detection/non-detection data *y*_*ij*_ observed at site *i* during survey *j* can be described as:

$$\frac{y_{ij}}{z_{i}∼Bernoulli\left(z_{i}\rho_{ij}\right)}$$ (2)

where *y*_*ij*_ is the observed ‘presence-absence’ data and *ρ*_*ij*_ is the probability of detection. We modelled the influence of covariates on occupancy and detectability using the logit link function in Eqs 1 and 2.

### Model fitting

Prior to model fitting, we identified seven species (Arnhem Land Rock Rat, Black Wallaroo, *Gehyra pamela*, *Pseudothecadactylus lindneri*, *Oedura gemmata*, Banded Fruit-dove, Chestnut-quilled Rock-pigeon) that have ranges restricted to the Arnhem Plateau, which is only a small fraction of the study region. We created a ‘plateau’ layer as a proxy for this geographic range by placing a 5 km buffer around the Arnhem escarpment, and only used data collected from within this area when fitting models. This meant that our estimates of occupancy were relative to the expected geographic range of species rather than to all sites in the study region, with predictions of the seven species above confined to our plateau layer.

We fitted occupancy and detectability models using the *unmarked* package [31] in R [32]. We assumed that occupancy could depend on any combination of environmental predictor variables, while detectability was modelled as a function of the sampling method (for those species with multiple detection methods), time since fire, fire frequency, and terrain ruggedness (Table 1). Our rationale for including these variables in the detectability component of the model is that we expected fire to influence vegetation structure and composition at sites and therefore possibly effect the ability of observers to sight individuals. Terrain ruggedness might also influence how well individuals can be sighted during surveys by limiting the line of sight of the observer.

We appended the detection histories for each species and modelled the sampling method in *unmarked* as an observational-level factor. We assumed a closed population over the survey period or that movement of species with large home ranges to and from sites was random [33]. We allowed for quadratic terms in all covariates in the occupancy model except for fire extent and fire patchiness, but did not expect any quadratic terms in the detectability component. All covariates were centred and standardised prior to model fitting [34].

We fitted the most complex model to each species and considered all possible combinations of covariates using the *pdredge* function in the R package *MuMIn* [35]. To avoid over-fitting, we constrained the number of covariates in each model at a ratio of 1:10 with the number of sites where a species was detected [36]. We ranked all models by their AIC value [37] and retained only those with a ΔAIC of less than six [38].

### Model selection

For each species, we screened our competing set of top models for ecological realism. Reviewing and scrutinizing models so that predictions conform to expert opinion or beliefs is generally encouraged in the species distribution modelling literature [39]. To aid this validation process we generated occupancy maps by predicting the top models to covariate raster layers mapped at 1 km resolution (S1 Fig). We generated 1 km maps from our original 100 m raster layers by reclassifying each covariate layer. Re-sizing rasters was done simply to reduce the image size of each map (<20 MB) for ease of use.

Occupancy maps were screened and discarded if: 1) there was perceived to be a severe mismatch between predicted occupancy and current beliefs about where the species occurs; or, 2) if predicted occupancy unrealistically approached 0 and/or 1 (i.e., boundary estimates), which can result from too few detections [40]. Consideration was given to species with very low occupancy and/or detectability (<0.1) as such estimates should be treated with caution [30]. Models with the highest AIC were accepted as the best model unless occupancy maps failed the validation process, in which case the next-best ranked model was considered.

We assessed model fit of the top-ranked model with sum of squared errors, Freeman-Tukey Chi-squared test, and Pearson’s Chi-squared test [41]. Goodness-of-fit tests were conducted with 1000 simulations. If the top-ranked model for a species failed any goodness-of-fit test it was discarded for the next-best ranked model. If we could not fit a model containing one or more covariates, we modelled constant occupancy across the study area with the sampling method as an observational-level factor (for those species with multiple detection methods). For each species, we calculated the mean occupancy and detectability for each method by averaging across estimates at sampling sites.

### Mapping species occupancy and richness

For species containing covariates in their ‘best-model’, we generated occupancy maps across the study region by predicting occupancy to mapped covariate raster layers at 1 km resolution (S1 Fig). Rather than predict occupancy to the entire study area, we limited our predictions to the mainland because offshore islands are disjunct locations where species might respond differently to the environmental covariates. We were also cautious about predicting occupancy well-outside the range of the sampled environmental domain. After comparing the environmental domain at monitoring sites (i.e., range of predictor covariate values) with that of the study area we masked out regions with much higher maximum temperature and fire patchiness, and much lower annual rainfall and vegetation cover, than what was sampled. This restricted our occupancy predictions in some parts of the study region, particularly in the south.

We built relative species richness maps by stacking occupancy maps for those birds, mammals, and reptiles for which we could fit models, and summed the predicted occupancy value in each cell across species [42]. We did not include species with constant occupancy (i.e., null models, or no environmental predictor variables in the model) and note that our species richness maps do not represent true species richness for the study area, but rather the richness of the species we could fit models to.

## Results

### Occupancy

**Fig 2:**
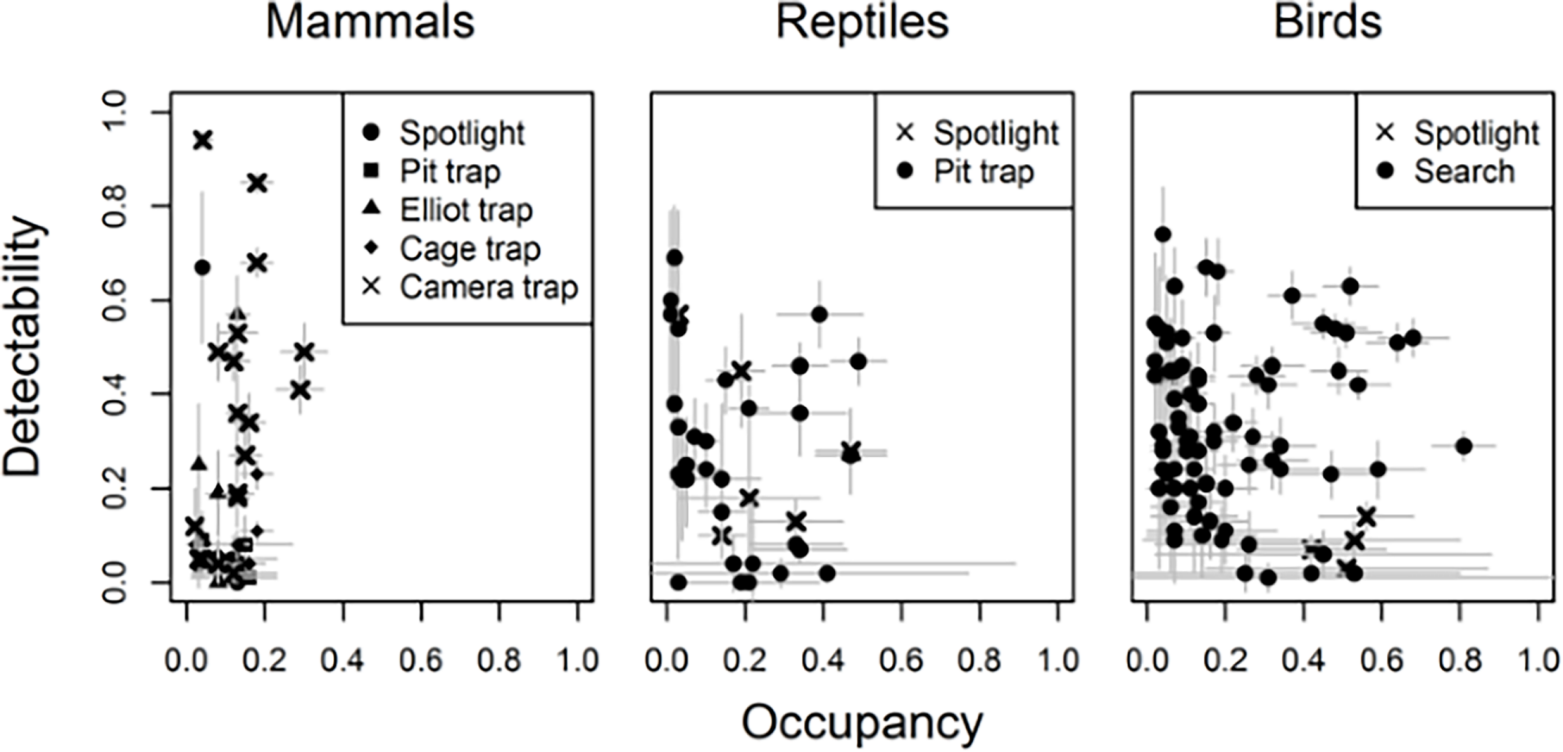
Occupancy versus detectability for mammals, reptiles and birds in Northern Australia. Occupancy (x-axis) versus detectability (y-axis) for mammals, reptiles and birds ± SE (grey bars) estimated with repeat detection/non-detection data collected at 333 sites in Northern Australia between 2011–2016. Different shapes represent the different sampling methods. Detectability for live trapping and spotlighting is estimated over the period of one day/night, while estimates of camera trapping is over the period of one week.

A total of 242 native species (147 birds, 69 reptiles and 26 mammals) were recorded during surveys of eight protected areas (S3–S5 Tables), representing 66% of the total number of bird, mammal and reptile species sampled over 20 years of the Three Parks Program. We fitted occupancy-detection models to 65% of these, of which a further 9% were discarded because they were considered implausible (S6–S9 Tables). This resulted in occupancy-detection models for 136 species, including 83 birds, 33 reptiles, and 20 mammals. Birds exhibited the most variability in mean occupancy across sampling sites, ranging from 0.02 ± 0.01 (Arafura Fantail) to 0.81 ± 0.08 (White-bellied Cuckoo-shrike) (Fig 2; S6 Table), while reptiles ranged from 0.01 ± 0.01 (*Ctenotus storri*) to 0.49 ± 0.07 (*Carlia amax*) (Fig 2; S7 Table). By comparison, the range in mean occupancy for mammals was lower than birds or reptiles, ranging from 0.02 ± 0.01 (Northern Quoll) to 0.30 ± 0.06 (Agile Wallaby) (Fig 2; S8 Table).

**Fig 3:**
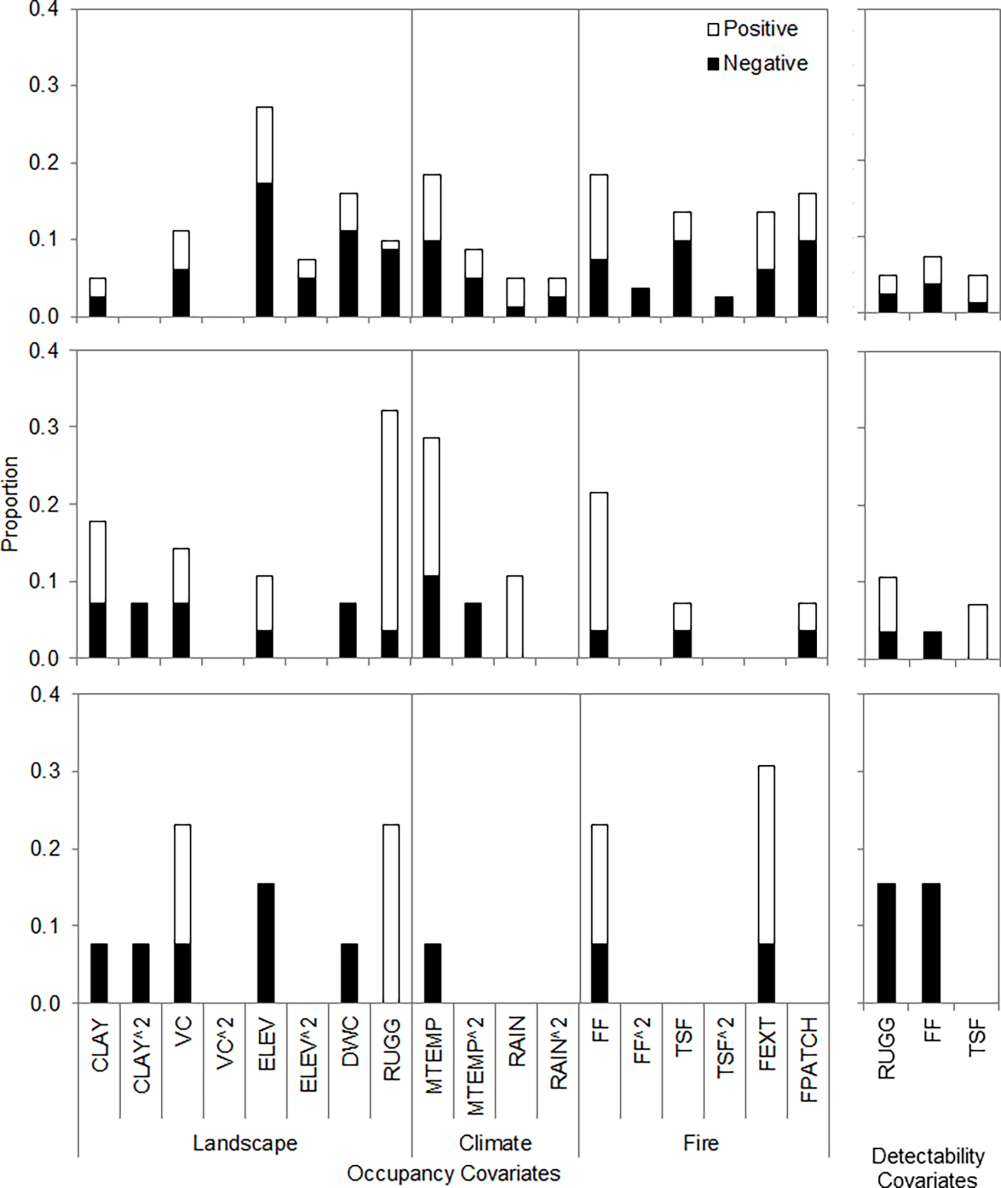
Environmental covariates commonly retained in occupancy and detectability models. The proportion of times (y-axis) each covariate (x-axis) was included in occupancy and detectability models for birds (top row), reptiles (middle row) and mammals (bottom row). Site covariates are grouped by landscape, climate or fire. Dark shading indicates the proportion of species that responded negatively to covariates, white shading indicates the proportion that responded positively.

**Fig 4:**
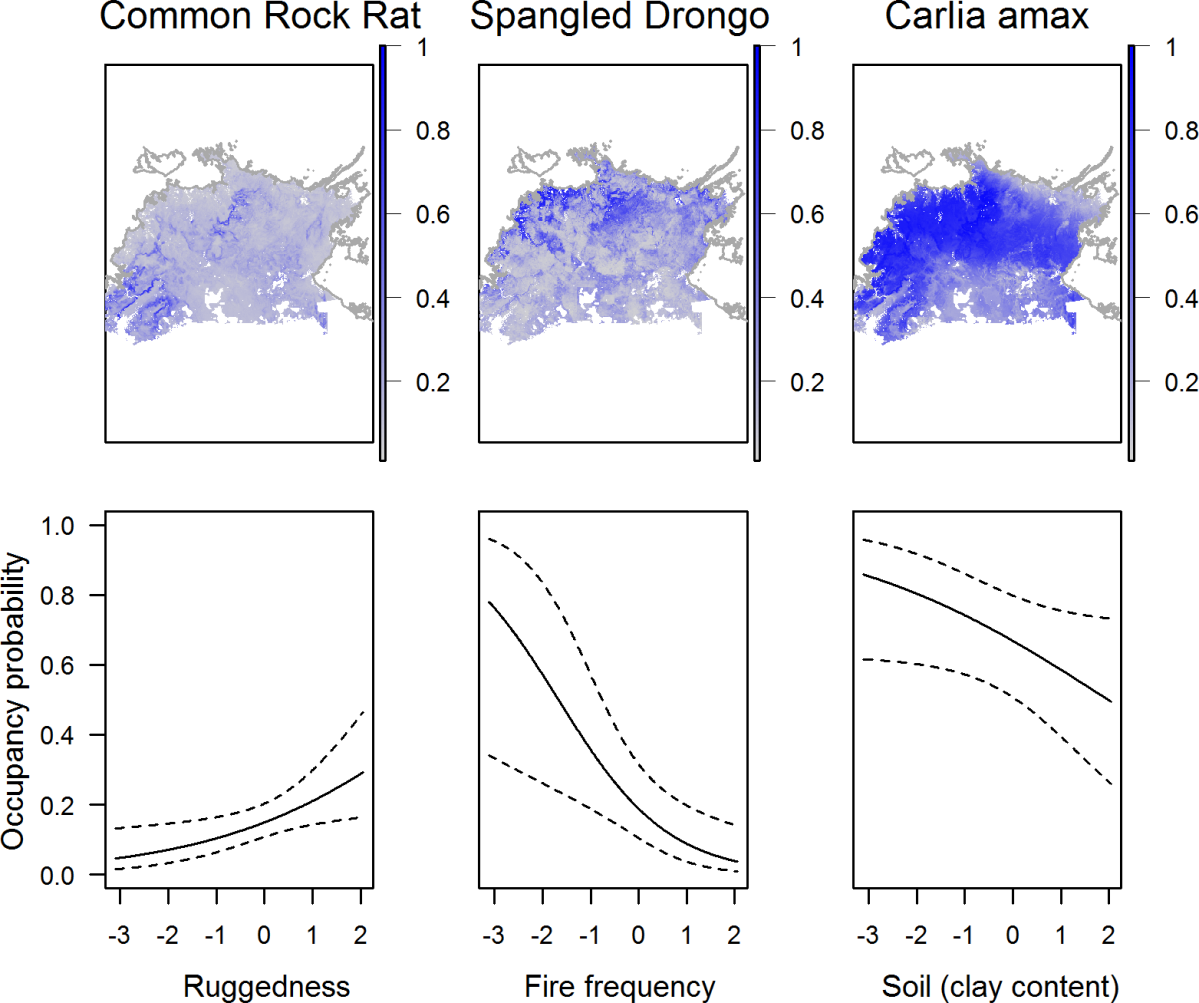
Relationship of covariates with occupancy for a selection of species. Example of three maps of predicted occupancy for Common Rock Rat (top left) Spangled Drongo (top centre) and *Carlia amax* (top right) across the Top End of Australia. The relationship between the probability of occupancy and selected covariates (scaled) for these three species are presented in the bottom row. Dotted lines represent 95% confidence intervals.

For birds, elevation was retained in 34% of occupancy models, followed by maximum temperature (27%), fire frequency (22%), fire patchiness (16%), distance to watercourse (16%), fire extent (13%), and time since fire (13%, Fig 3). For reptiles, maximum temperature was retained in 35% of occupancy models, followed by ruggedness (32%), clay content (25%), and fire frequency (21%, Fig 3). For mammals, fire extent was the covariate most commonly included in occupancy models (33%), followed by fire frequency and vegetation cover (both 25%), ruggedness and elevation (both 18%, Fig 3). Of the 136 modelled species, 121 included one or more covariates (S2 Fig). Maps of predicted occupancy and the response of occupancy to covariates are shown for a selection of species in Fig 4, and occupancy maps for all species are presented in S3–S5 Figs.

### Detectability

Mean detectability during a day/night of live trapping (mammals and reptiles) or day/night of searches (birds, mammals and reptiles) was lowest for mammals (0.13 ± 0.05), and higher for birds (0.23 ± 0.07) and reptiles (0.28 ± 0.10), although estimates varied widely between species and between sampling methods (Fig 2; S6–S8 Tables). For example, daily detectability of birds (three surveys) ranged from 0.01 ± 0.03 (Collared Sparrowhawk) to 0.74 ± 0.01 (Rufous-banded Honeyeater, S6 Table). Nightly detectability of birds using spotlighting (one survey) was lower, ranging from 0.03 ± 0.02 (Barking Owl) to 0.44 ± 0.21 (Spotted Nightjar). For reptiles, detectability per day/night using the array of pitfall traps ranged from 0.02 ± 0.02 (*Heteronotia planiceps*) to 0.69 ± 0.11 (*Ctenotus piankai*; S7 Table), whereas detectability during a night of spotlighting (one survey) ranged from 0.10 ± 0.05 (*Gehyra nana*) to 0.57 ± 0.22 (*Oedura marmorata*). Of three reptiles detected using both methods, two were more detectable by spotlighting (S7 Table).

Detection probability for mammals using a night of pitfall trapping was very low, ranging from 0.01 ± 0.01 (Grassland Melomys) to 0.09 ± 0.06 (Red-cheeked Dunnart) (Fig 2; S8 Table). In contrast, nightly detectability using spotlighting varied widely from 0.03 ± 0.03 (Sandstone Antechinus) to 0.67 ± 0.16 (Sugar Glider). Similarly, nightly detectability using the array of Elliott traps ranged widely, from 0.01 ± 0.01 (Delicate Mouse) to 0.57 ± 0.08 (Arnhem Land Rock Rat). The Arnhem Land Rock Rat had the lowest detection probability using cage traps (0.02 ± 0.02), while the Northern Brown Bandicoot had the highest (0.23 ± 0.03). Weekly detectability using the array of camera traps ranged from 0.02 ± 0.02 for the Delicate Mouse to 0.94 ± 0.06 for Short-eared Rock Wallaby. Of the 20 mammals, six were detected exclusively on camera traps (mainly macropods), two were not detected using this method, and 13 were detected by cameras and another method. Of these 13 mammals, five were detected with a higher probability on cameras deployed for one week than when using nighty live trapping methods.

Fire frequency was retained in 7% of bird, 3% of reptile, and 16% of mammal detectability models, with an exclusively negative effect on reptile and mammal detectability, and mixed effect on birds (Fig 3; S9–S11 Tables). Ruggedness was retained in 5% of bird, 10% of reptile, and 16% of mammal detectability models, with an exclusively negative effect on mammals, and a mixed effect on birds and reptiles. Time since fire was retained in relatively few detectability models.

### Species richness

**Fig 5:**
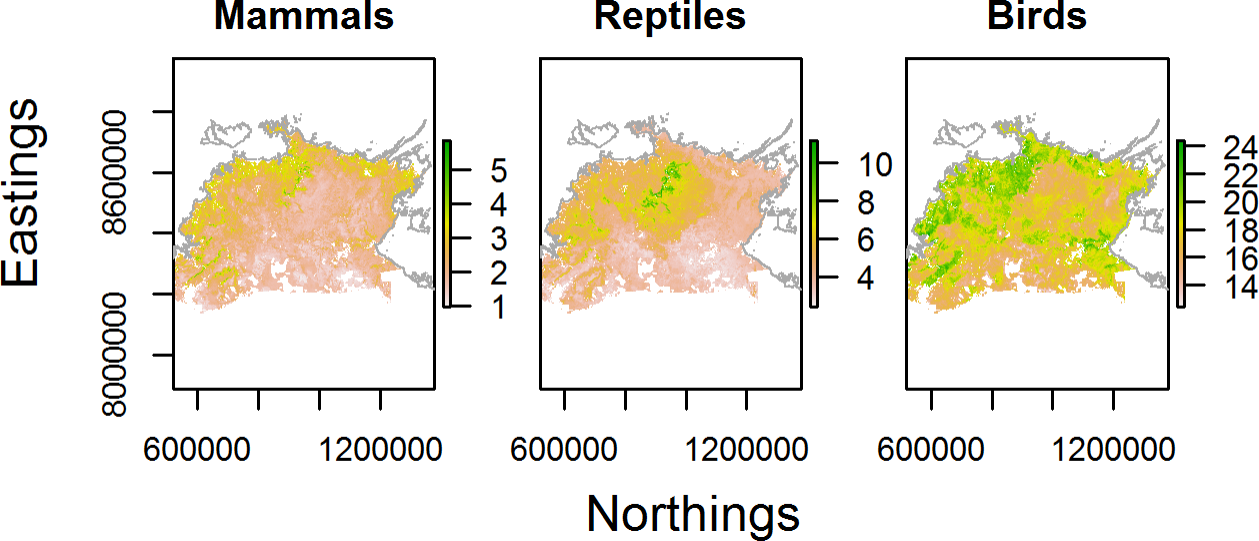
Relative species richness maps. Richness maps for 20 mammals, 33 reptiles, and 84 birds across the Top End of northern Australia. Note, the scale bar is the summed probability of occupancy for each species, not the expected number of species, and richness refers to the pool of modelled species, not all species in the region.

For mammals, our relative species richness map predicted higher richness in rugged terrain along the edge of the Arnhem plateau and in rugged areas in the south west, as well as across coastal lowlands in the north and west (Fig 5). This pattern largely reflects the influence of terrain ruggedness and vegetation cover on predicted occupancy for many of the mammals (Fig 3; S1 Fig). Relative reptile richness was predicted to be highest on the Arnhem plateau, and in fragmented areas of rugged terrain to the south west of the study region (Fig 5). In contrast, relative bird richness was highest across vast coastal and lowland areas due to the combination of several landscape and fire covariates (Figs 3 and 5; S1 Fig).

## Discussion

Few studies have modelled the distribution of birds [43], mammals [24] or reptiles across the Top End of Australia, or compared the relative effectiveness of multiple sampling methods at detecting species during monitoring (e.g., south-eastern Australia [18]). In this study, we fitted occupancy-detection models to over half of the species recorded during surveys in eight conservation reserves, and predicted relative species richness across an extensive area of the Top End of northern Australia. To our knowledge, this is the first attempt to: 1) generate broad-scale high resolution species occupancy and richness maps for a suite of species while accounting for imperfect detection; 2) model the distribution of reptiles across the Top End in response to a range of climatic, landscape and fire variables; and, 3) provide a comprehensive comparison of the relative effectiveness of commonly used survey methods for vertebrates in the region. This information is an important first step for evaluating and re-designing the Three Parks Program in the Top End.

### Influence of environmental covariates on occupancy

For mammals, fire and landscape variables were retained in a higher proportion of occupancy models than broad-scale climatic variables. It should be noted, however, that we fitted occupancy models to fewer mammals compared to other species groups, which meant that the relative contribution of covariates in percentage terms was highly sensitive to the choice of model for each mammal species. There is mounting evidence that contemporary mammal declines in northern Australia have been influenced by fire frequency [44] and extent [12]. Causal factors driving species distributions are not always evident from observed patterns of occupancy, and are more reliably demonstrated with studies of spatial dynamics (e.g., dynamic occupancy modelling [33]). Nonetheless, we observed the anticipated negative association between fire frequency and extent and occupancy for several mammal species [12,45]. However, positive associations also occurred for some mammals such as Antilopine Wallaroo and Black-footed Tree-rat. Higher occupancy in burnt areas may not necessarily be indicative of a species preference for those conditions, but an artefact of species being restricted to these regions due to other biotic processes not considered in our study. For example, Black-footed Tree-rat occur in lowland woodlands, a habitat type more prone to extensive fires, thereby contributing to the observed positive relationship between fire extent and occupancy.

Higher predicted mammal richness in rugged terrain followed by coastal lowlands to the north and west is in broad agreement with current hypotheses about where mammals are persisting in the Top End. Monitoring and distribution data indicate that range contractions have occurred from south to north, with earlier and more severe mammal declines inland [13], and greater resilience of populations of some species on Coburg Peninsula and offshore islands [46]. Previous studies concur with our findings of high mammal richness in rugged/rocky areas in the region [47]. Rocky upland areas contain a largely distinct mammal assemblage (e.g., rock rats and rock wallabies), with Arnhem Land Rock Rat and Black Wallaroo endemic to the Arnhem plateau. Mammals in rugged terrain may be able to persist longer as factors driving declines elsewhere may be diminished or absent in these areas. For example, rocky and rugged terrain presents barriers to fire, resulting in patchier and less damaging fires compared to less complex lowland areas where there are generally fewer impediments to fire spread [48]. While landscape variables were retained in many of our models, mammal declines are thought to be driven by more complex interactions between abiotic and biotic processes, such as altered fire regimes [12], predation by feral cats and/or grazing by feral herbivores [10,13], and the interaction of these factors [49,50], as well as interaction with rainfall and productivity [47]. Accurately accounting for and incorporating abiotic and biotic interactions into predictive models requires a good understanding of these ecological processes.

Unlike birds, most studies of reptile distributions across northern Australia have focused on the influence of habitat variables at finer scales than we used in our analysis [23,51], however, these studies do not attempt to predict distributions across landscapes. One study within Kakadu National Park considered 102 species and found that most were associated with a gradient of substrate and moisture availability, but rarely vegetation structure, with highest richness in rocky areas [23]. The results of our broader scale study concur with these findings by identifying high reptile richness in rugged areas–a useful proxy for rockiness–despite the fact that we only modelled a subset of the reptile community. Another study that sampled across the rainfall gradient of the Top End found that sites in high rainfall areas supported the most reptile species, and sites on clay soils supported fewer species [25]. Our modelling approach found little support for rainfall as a predictor of occupancy, however, clay content was retained in more reptile models than for mammals and birds, albeit still a small proportion.

In comparison to mammals and reptiles, relative bird richness is predicted to be highest across a large proportion of the study region due to the broad distributions of many birds throughout northern Australia [52]. The relatively high contribution of maximum temperature and fire frequency on bird occupancy is largely in agreement with a broad-scale study by Reside et al. [43], who found that temperature seasonality and rainfall was the greatest predictor of occurrence for 44 species across northern Australia, with a small, yet significant effect of fire frequency for some species. Several studies have found marginally higher diversity in higher rainfall areas across the region, by inferring distribution patterns of birds from site-based sampling [53]. Generalisations about the influence of rainfall and climate on species distributions are of limited value in isolation, because as we have demonstrated, occurrence may vary extensively within these gradients due to finer-scales factors such as soil, vegetation cover, and fire.

### Detectability using traditional sampling methods

Unsurprisingly, we found high variability in the detection probability of birds, reptiles, and mammals using our standardised monitoring protocol. Contrary to our expectations, fire frequency had a negative influence on the detectability of mammals, reptiles, and birds, while ruggedness had both positive and negative effects on detectability. The role of site covariates such as these on detectability therefore requires further consideration using finer-scale covariate data. Camera trapping was relatively effective at detecting mammals compared with live trapping, which agrees with recent research for similar species [17]. Importantly, the effectiveness of camera trapping seemingly increased with mammal body size (e.g., Delicate Mouse 0.02 ± 0.02 versus Agile Wallaby 0.49 ± 0.06). This result highlights that camera traps should not be used exclusively if the goal is to detect ground-dwelling mammals of all sizes. However, given cameras can be left unattended at sites for long periods they may present a relatively cost-effective sampling method compared with live trapping, although cameras need to be retrieved and substantial effort is required to process photos. Evaluating the cost-effectiveness of competing approaches to surveying species, taking into account the trade-off between effort/cost and precision of the estimates of occupancy [54,55], would benefit the redesign of the Three Parks Program.

### Limitations

We made several assumptions that may have influenced occupancy and detectability estimates. Firstly, the initial set of environmental covariates considered in the most complex models were identical for birds, reptiles and mammals. Alternatively, we could have specified unique models for each species group, or for each species, however, this would have increased the complexity of our analysis. Secondly, we ignored within year variability in detection probability or any differences in detectability between observers. Expanding our detectability models to test for observer and seasonal effects warrants further investigation. Thirdly, a mismatch in the initial resolution of covariate raster layers may have introduced uncertainty into the analysis. Finer-resolution mapping of the coarsely mapped covariates, particularly fire, will likely improve our understanding of their relationship with occupancy and detectability. Finally, the home range of species varied considerably relative to the area of surveyed sites, which influences our interpretation of occupancy. For species with home ranges similar in size to the sampling unit of 100 x 100 m (i.e. reptiles and small mammals), occupancy can be interpreted as the probability of a species being present at a site. However, many of the birds and large mammals will likely have home ranges much larger than this area, meaning occupancy for these species is better interpreted as probability of use. This assumes that the lack of closure between repeat surveys is random with respect to movement in and out of sites [33].

There are several caveats with our relative species richness maps. Firstly, species richness maps were generated by summing the mean predicted occupancy across species. This approach ignores the variance around each species occupancy estimate, assumes all species are given equal weight, and can potentially over-predict richness at species-poor sites and under-predict richness at species-rich sites [42]. Secondly, for numerous species we were limited to presenting only null (constant) models which did not contribute to richness maps, and models with single covariates or with associated low regression coefficients. These models may be deemed as having limited value for interpreting patterns of occupancy; however, they were retained as they present a starting point from which we can build upon as data becomes available. Thirdly, we could only fit models to 136 species, which is approximately half of the species recorded at sampling sites, and only a fraction of the ~600 terrestrial vertebrates known to occur in the region. This means our richness maps are biased towards the more common, widespread and detectable species, and do not represent the true richness of the study region. Very few threatened species contributed to our species richness maps because there were either too few detections or the models were deemed implausible (due to inadequate predictors or relatively few detections). Updating our models in the future using data from new or existing monitoring sites might improve the representation of threatened species in the species richness maps. Alternatively, occupancy maps for these species could be developed using alternative data (i.e. presence-only data) and modelling approaches. This is an interesting avenue of further research [56,57] but beyond the scope of this study.

We generated relative occupancy and species richness maps by predicting our models to environmental covariates mapped across the Top End. In doing so, we extrapolated our predictions outside the geographic area that was sampled. This is common practice in species distribution modelling, provided care is taken to avoid extrapolating to sites where the response of species to environmental covariates might differ [58]. We were cautious when extrapolating our predictions to not predict far outside the environmental domains (i.e., combination of predictor ranges) that were sampled. Furthermore, we did not predict occupancy to offshore islands as obvious biogeographical barriers may have prevented dispersal, and islands are geographically disjunct locations where species might respond differently to the environmental covariates studied. To assess how well our models matched the observed process, we ran goodness-of-fit tests (Sum of Squared Errors, Freeman-Tukey Chi-squared, and Pearson’s Chi-squared test) on the best models for all species. We could not use traditional measures of predictive performance, such as AUC, given our non-detections were not confirmed absences, due to imperfect detection [59]. Further research into goodness-of-fit testing and model validation is needed in the context of occupancy-detection models.

The utility of models as a planning tool in conservation is influenced by the selection of relevant predictors [39]. Our models were constrained by using only 11 predictors, and the absence of numerous others well known to drive species occurrence and distributions, but for which broad scale mapping is unavailable (e.g., cat occurrence, grazing pressure, habitat structure and complexity, etc.). In the absence of a definitive set of mapped covariates there are two options available to us when designing monitoring. (1) Not using a spatial model–this ignores not only the factors driving distribution that can’t be mapped, but all spatially mappable factors. Instead, we choose to use imperfect models to improve the design of monitoring acknowledging there are deficiencies. (2) Using mappable environmental predictors as ‘distal predictors’ [60] or surrogates of the actual proximal drivers of distribution. The use of ‘distal predictors’ requires prior knowledge of the interaction between biotic factors, or biotic and abiotic factors. For example, the abundance of feral cats or feral herbivores in some parts of northern Australia is known to be influenced by climate, topography and vegetation type [61]. Where these correlations exists, the estimated response of species to these environmental factors will incorporate these effects, albeit imperfectly, thereby acting as distal predictors of feral cats and stock in this example. When a map of cat and grazing pressure distribution is available at sufficiently high spatial resolution, it would make sense to incorporate them into the analysis.

### Informing monitoring decisions

Our species-level occupancy and detectability modelling combined with our species richness maps will inform redesign of the Three Parks Program. Firstly, our occupancy and species richness maps can provide a starting point from which to assess how existing or proposed monitoring sites align with species distributions. Decision-makers can then consider how new sites could be positioned to maximise either the spatial coverage of species or the number of species detected at sites. Furthermore, the location of sites can be compared with the expected or known drivers of occupancy to ensure adequate sampling across gradients, and relocated if necessary to stratify more effectively, thereby improving the ability to study faunal responses those drivers. For example, comparison between covariate values at sampled sites with values across the entire study region in this study suggests that the existing sites are fairly representative of some covariates (S6 Fig; e.g., topographic ruggedness, clayiness, fire extent), but do not sample the full spectrum of others (e.g., fire patchiness, annual rainfall, vegetation cover)—warranting the need to truncate our predictions of occupancy and richness to the environmental domain for which we had data (see methods). Thus, a redesigned vertebrate monitoring program for the Top End might seek to improve monitoring in regions masked out of our occupancy predictions (in the south of the study region or on islands) to improve our understanding of species across these environmental gradients.

Our detectability estimates for the six sampling methods will help evaluate which methods are worth retaining in the revised Three Parks program. Detectability estimate will also inform how much sampling effort is required to detect species with a given level of confidence. The results presented here can also inform similar monitoring decisions for related species outside of the study region that lack rigorous detectability estimates. An added benefit of estimating occupancy and detectability is that the statistical power of a revised monitoring program can be assessed. Statistical power is related to the number of sites, initial occupancy, detectability, the effect size, and the Type I error rate [62]. Future work could use the occupancy and detectability values reported here to quantify how many sites, or how much survey effort is required to detect changes in species occupancy with sufficient power (e.g., [5,6]).

Our work demonstrates that high resolution, broad-scale occupancy-detection modelling can be achieved using repeat presence-absence data that accounts for imperfect detection. Modelling repeat presence-absence data is an advance on the more widely used presence-only models (commonly fitted with the Maxent software, e.g., [24,43]) for a range of reasons, including explicitly accounting for ‘false zero’ observations in survey data. This is important because ignoring false absences can lead to biased inferences about species distributions and over-confident precision around parameter estimates [33,63,64]. Importantly, accounting for imperfect detection also allows one to determine whether an observed difference in occupancy over space and time is because of changes in populations, or because of a change in the detection probability. Despite these benefits, only a small minority of studies measuring species richness, occupancy or range/distribution account for imperfect detection [2,4]. Our study demonstrates that this approach can be applied to a broad suite of species across relatively large spatial scales.

## Conclusion

The value of occupancy and detectability models is that they allow inference and prediction based on multiple, additive effects at multiple scales, thereby providing a solid basis for the spatial and temporal allocation of monitoring effort. We provided a snapshot of the current occupancy and detectability for a large proportion of the mammal, reptile and bird community across the Top End of northern Australia at a scale relevant for informing the redesign of a long-running monitoring program. Our results indicate where vertebrate species are likely to occur with respect to existing monitoring sites, and high variability in detectability between species and sampling methods. This information is crucial to the design of future monitoring programs across the Top End of northern Australia.

## Supporting information

S1 Fig Mapped climatic, topographic and fire covariates used to model species occupancy and detectability. Covariates (scaled) at 1 km resolution used to model occupancy and detectability of 242 birds, mammals and reptiles recorded at 333 sites across the Top End of northern Australia.
(TIF)

S2 Fig Number of covariates included in occupancy and detectability models for 136 species. Proportion of models per animal group with 1–8 covariates included in the best model. Note, maximum covariate count for detectability models is 3, and method was included in detectability models for species with multiple methods of detection, but was not included in the covariate count presented here.
(TIF)

S3 Fig Mammal occupancy maps across the Top End of northern Australia. Occupancy maps for mammals with covariates in the best model. Light grey represents zero occupancy, while blue represents an occupancy probability of 1.
(TIFF)

S4 Fig Reptile occupancy maps across the Top End of northern Australia. Occupancy maps for reptiles with covariates in the best model. Occupancy maps for reptiles with covariates in the best model. Light grey represents zero occupancy, while blue represents an occupancy probability of 1.
(PDF)

S5 Fig Bird occupancy maps across the Top End of northern Australia. Occupancy maps for birds with covariates in the best model. Occupancy maps for birds with covariates in the best model. Light grey represents zero occupancy, while blue represents an occupancy probability of 1.
(PDF)

S6 Fig Sampled environmental domains across the Top End of northern Australia. Frequency histograms of covariate values occurring within the truncated (i.e., not the full extent of the Top End) mapping region, showing the representativeness of sampling sites for birds (red), mammals (blue), and reptiles (orange) in comparison to the spectrum of environmental conditions to which species occupancy was predicted.
(TIFF)

S1 Table Sampling methods applied at monitoring sites across the eight conservation reserves. Summary of the method of detection pooled to generate detection histories per animal group per location: Djelk Indigenous Protected Area (DIPA); Fish River Station (FRS); Garig Gunak Barlu National Park (GGBNP); Gregory National Park (GNP); Kakadu National Park (KNP); Litchfield National Park (LNP); Nitmiluk National Park (NNP); Warrdeken Indigenous Protected Area (WIPA). Note, species-specific detection histories were generated from a subset of all methods available per animal group.
(PDF)

S2 Table Pairwise correlation matrix for candidate predictor variable. Covariates with a Spearman’s correlation coefficient greater than 0.7 are shown in bold, with one of a pair excluded from the analysis.
(PDF)

S3 Table Model coefficients for the occupancy component of the reptile models. Model coefficients for the occupancy component of the reptile models. Note, species containing only dashes were recorded during surveys but were unable to be modelled.
(PDF)

S4 Table Model coefficients for the occupancy component of the bird models. Note, species containing only dashes were recorded during surveys but were unable to be modelled.
(PDF)

S5 Table Model coefficients for the occupancy component of the mammal models. Note, species containing only dashes were recorded during surveys but were unable to be modelled.
(PDF)

S6 Table Estimates of occupancy and detectability for each bird species. Occupancy and detectability estimates (over one day/night) for 83 birds modelled using diurnal active searches and spotlighting averaged across monitoring sites.
(PDF)

S7 Table Estimates of occupancy and detectability for each reptile species. Occupancy and detectability (over one day/night) estimates for 33 reptiles modelled using pitfall trapping and/or spotlighting data averaged across monitoring sites.
(PDF)

S8 Table Estimates of occupancy and detectability for each mammal species with alternative sampling methods. Occupancy and detectability for 20 mammals modelled using live trapping and spotlighting averaged across 326 sites, and camera trapping averaged across 168 sites in northern Australia. Detectability estimates for live trapping and spotlighting is over the period of a day/night, and one week for camera trapping. * denotes introduced species.
(PDF)

S9 Table Model coefficients for the detection component of the reptile models. Note, species containing only dashes were recorded during surveys but were unable to be modelled.
(PDF)

S10 Table Model coefficients for the detectability component of the bird models. Note, species containing only dashes were recorded during surveys but were unable to be modelled.
(PDF)

S11 Table Model coefficients for the detectability component of the mammal models. Note, species containing only dashes were recorded during surveys but were unable to be modelled.
(PDF)

## References

[1] Lindenmayer DB, Gibbons P, Bourke M, Burgman M, Dickman CR, Ferrier S, et al Improving biodiversity monitoring. Austral Ecology. 2012: 37:285–294.

[2] Yoccoz NG, Nichols JD, Boulinier T. Monitoring of biological diversity in space and time. Trends in Ecology and Evolution. 2001; 16:446–453.

[3] MacKenzie DI, Royle JA Designing occupancy studies: general advice and allocating survey effort. Journal of Applied Ecology. 2005; 42:1105–1114.

[4] Keller F, Swihart RK. Accounting for imperfect detection in ecology: a quantitative review PLoS ONE. 2014; 9(10): e111436 10.1371/journal.pone.0111436 25356904 PMC4214722

[5] Morrison LW. Assessing the Reliability of Ecological Monitoring Data: Power Analysis and Alternative Approaches. Natural Areas Journal. 2007; 27:83–91.

[6] Sewell D, Guillera-Arroita G, Griffiths RA, Beebee TJC. When Is a Species Declining? Optimizing Survey Effort to Detect Population Changes in Reptiles. PLoS ONE. 2012; 7(8): e43387 10.1371/journal.pone.0043387 22937044 PMC3425567

[7] Woinarski JCZ, Legge S, Fitzsimons JA, Traill BJ, Burbidge AA, Fisher A, et al The disappearing mammal fauna of northern Australia: context, cause, and response. Conservation Letters. 2011; 4:192–201.

[8] Woinarski JCZ, Armstrong M, Brennan K, Fisher A, Griffiths AD, Hill B, et al Monitoring indicates rapid and severe decline of native small mammals in Kakadu National Park, northern Australia. Wildlife Research. 2010; 37:116–126.

[9] Fisher DO, Johnson CN, Lawes MJ, Fritz SA, McCallum H, Blomberg SP, et al The current decline of tropical marsupials in Australia: is history repeating? Global Ecology and Biogeography. 2013: 23:181–190.

[10] Legge S, Kennedy MS, Lloyd R, Murphy SA, Fisher A. Rapid recovery of mammal fauna in the central Kimberley, northern Australia, following the removal of introduced herbivores. Austral Ecology. 2011; 36:791–799.

[11] Russell-Smith J. Edwards AC, Woinarski JCZ, Fisher A, Murphy BP, Lawes MJ. North Australian tropical savannas: The three parks savanna fire-effects plot network In: Burns E, Lindenmayer D, Lowe A, Thurgate N, editors. Biodiversity and Environmental Change: Monitoring, Challenges and Direction. Melbourne, AUS; CSIRO Publishing; 2014 p. 335–378.

[12] Lawes MJ, Murphy BP, Fisher A, Woinarski JCZ, Edwards AC, Russell-Smith J. Small mammals decline with increasing fire extent in northern Australia: evidence from long-term monitoring in Kakadu National Park. International Journal of Wildland Fire. 2015; 24:712–722.

[13] Ziembicki M, Woinarski JCZ, Webb J, Vanderduys E, Tuft K, Smith J, et al Stemming the tide: progress towards resolving the causes of decline and implementing management responses for the disappearing mammal fauna of northern Australia. Therya. 2015; 6:169–225.

[14] Wintle B. A., Kavanagh R. P., McCarthy M. A., and Burgman M. A. 2005 Estimating and dealing with detectability in occupancy surveys for forest owls and arboreal marsupials. Journal of Wildlife Management 69:905–917.

[15] Wintle BA, Walshe TV, Parris KM, McCarthy MA. Designing occupancy surveys and interpreting non-detection when observations are imperfect. Diversity and Distributions. 2012; 18:417–424.

[16] McDonald PJ, Griffiths AD, Nano CEM, Dickman CR, Ward SJ, Luck GW. Landscape-scale factors determine occupancy of the critically endangered central rock-rat in arid Australia: The utility of camera trapping. Biological Conservation. 2015; 191:93–100.

[17] Smith J, Legge S, James A, Tuft K. Optimising camera trap deployment design across multiple sites for species inventory surveys. Pacific conservation biology. 2017; 23:43–51.

[18] De Bondi N, White JG, Stevens M, Cooke R. A comparison of the effectiveness of camera trapping and live trapping for sampling terrestrial small-mammal communities. Wildlife Research. 2010 37:456–465.

[19] Einoder LD, Southwell DM, Gillespie GR, Fisher A, Lahoz-Monfort JJ. Wintle BA. Optimising broad-scale monitoring for trend detection: review and re-design of a long-term program in northern Australia In: Legge S, Lindenmayer D, Robinson N, Scheele B, Southwell D, Wintle B., editors. Monitoring Threatened Species and Ecological Communities. Melbourne, AUS: CSIRO Publishing: 2018 p. 165–178.

[20] Williams RJ, Duff GA, Bowman D, Cook GD. Variation in the composition and structure of tropical savannas as a function of rainfall and soil texture along a large-scale climatic gradient in the Northern Territory, Australia. Journal of Biogeography. 1996; 23:747–756.

[21] Bowman D, Brown GK, Braby MF, Brown JR, Cook LG, Crisp MD, et al Biogeography of the Australian monsoon tropics. Journal of Biogeography. 2010 37:201–216.

[22] Gillespie GR, Brennan K, Gentles T, Hill B, Low Choy J, Mahney T, et al A guide for the use of remote cameras for wildlife surveys in northern Australia National Environmental Research Program, Northern Australia Hub, Charles Darwin University, Casuarina, NT; 2015.

[23] Woinarski JCZ, Gambold N. Gradient analysis of a tropical herpetofauna—distribution patterns of terrestrial reptiles and amphibians in stage II of Kakadu National Park, Australia. Wildlife Research.1992; 19:105–127.

[24] Milne DJ, Fisher A, Pavey CR. Models of the habitat associations and distributions of insectivorous bats of the Top End of the Northern Territory, Australia. Biological Conservation. 2006 130:370–385.

[25] Woinarski JCZ, Fisher A, Milne D. Distribution patterns of vertebrates in relation to an extensive rainfall gradient and variation in soil texture in the tropical savannas of the Northern Territory, Australia. Journal of Tropical Ecology. 1999; 15:381–398.

[26] Firth RSC, Woinarski JCZ, Brennan KG, Hempel C. Environmental relationships of the brush-tailed rabbit-rat, *Conilurus penicillatus*, and other small mammals on the Tiwi Islands, northern Australia. Journal of Biogeography. 2006 33:1820–1837.

[27] Guerschman JP, Hill MJ, Renzullo L, Barrett DJ, Marks AS, Botha E. Estimating fractional cover of photosynthetic vegetation, non-photosynthetic vegetation and base soil in the Australian tropical savanna region upscaling the EO-1 Hyperion MODIS sensors. Remote Sensing of Environment. 2009; 113:928–945.

[28] Braithwaite RW. Effects of fire regimes on lizards in the wet-dry tropics of Australia. Journal of Tropical Ecology. 1987; 3:265–275.

[29] Dormann CF, Elith J, Bacher S, Buchmann C, Carl G, Carre G, et al Collinearity: a review of methods to deal with it and a simulation study evaluating their performance. Ecography. 2013; 36:27–46.

[30] MacKenzie DI, Nichols JD, Lachman GB, Droege S, Royle J, Langtimm CA. Estimating site occupancy rates when detection probabilities are less than one. Ecology. 2002; 83:2248–2255.

[31] Fiske I, Chandler R. unmarked: An R Package for Fitting Hierarchical Models of Wildlife Occurrence and Abundance. Journal of Statistical Software. 2011; 43:1–23.

[32] R Development Core Team. R: A language and environment for statistical computing. R Foundation for Statistical Computing, Vienna, Austria 2014.

[33] MacKenzie DI, Nichols JD, Royle JA, Pollock KH, Bailey LL, Hines JE. Occupancy Estimation and Modelling: Inferring Patterns and Dynamics of Species Occurrence. Burlington: Elsevier/Academic Press; 2006.

[34] Gelman A, Hill J. Data analysis using regression and multilevel/hierarchical models New York: Cambridge University Press; 2006.

[35] Barton K. MuMIn: multi-model inference. R package version 1.15.1, R package version 2013.

[36] Harrell FE. Regression modelling strategies: with application to linear models, logistic regression, and survival analysis 1st Edition edition New York: Springer: 2001.

[37] Akaike H. Information theory and an extension of the maximum likelihood principle In: Parzen E, Tanabe K, Kitagawa G. editors. Selected Papers of Hirotugu Akaike. Springer Series in Statistics (Perspectives in Statistics). New York, NY: Springer US: 1998 p. 199–213.

[38] Burnham KP, Anderson DR. Model selection and inference: a practical information-theoretic approach. New York: Springer-Verlag; 1998.

[39] Elith J, Lethwick J. The contribution of species distribution modelling to conservation prioritization In. Moilanen A, Wilson K, Possingham H. editors. Spatial Conservation Prioritization: Quantitative Methods and Computational Tools. Oxford University Press 2009.

[40] Guillera-Arroita G, Ridout MS, Morgan BJT. Design of occupancy studies with imperfect detection. Methods in Ecology and Evolution. 2010; 1:131–139.

[41] MacKenzie DI. Bailey LL. Assessing the fit of site-occupancy models. Journal of Agricultural Biological and Environmental Statistics. 2004; 9:300–318.

[42] Calabrese JM, Certain G, Kraan C, Dormann CF. Stacking species distribution models and adjusting bias by linking them to macro-ecological models. Global Ecology and Biogeography. 2014; 23:99–112.

[43] Reside AE, VanDerWal J, Kutt A, Watson I, Williams S. Fire regime shifts affect bird species distributions. Diversity and Distributions. 2012; 18:213–225.

[44] Radford IJ, Gibson LA, Corey B, Carnes K, Fairman R. Influence of Fire Mosaics, Habitat Characteristics and Cattle Disturbance on Mammals in Fire-Prone Savanna Landscapes of the Northern Kimberley. PLoS ONE. 2015; 10(6): e0130721 10.1371/journal.pone.0130721 26121581 PMC4488076

[45] Andersen AN, Cook GD, Corbett LK, Douglas MM, Eager RW, Russell-Smith J, et al Fire frequency and biodiversity conservation in Australian tropical savannas: implications from the Kapalga fire experiment. Austral Ecology. 2005: 30:155–167.

[46] Davies HF, McCarthy MA, Firth RSC, Woinarski JCZ, Gillespie GR, Andersen AN, et al Top-down control of species distributions: feral cats driving the regional extinction of a threatened rodent in northern Australia. Diversity and Distributions. 2017; 23:272–283.

[47] Woinarski JCZ. Biogeography and conservation of reptiles, mammals and birds across North-western Australia: an inventory and base for planning an ecological reserve system. Wildlife Research. 1992; 19:665–705.

[48] Bradley AJ, Kemper CM, Kitchener DJ, Humphreys WF, How RA. Small mammals of the Mitchell Plateau Region, Kimberley, Western Australia. Australian Wildlife Research. 1987: 14:397–41.

[49] Leahy L, Legge S, Tuft K, McGregor HW, Barmuta LA, Jones ME, et al Amplified predation after fire suppresses rodent populations in Australia's tropical savannas. Wildlife Research. 2015; 42:705–716.

[50] McGregor H, Legge S, Jones ME, Johnson CN. Feral Cats Are Better Killers in Open Habitats, Revealed by Animal-Borne Video. PLoS ONE. 2015; 10(8): e0133915 10.1371/journal.pone.0133915 26288224 PMC4545751

[51] Price B, Kutt AS, McAlpine CA. The importance of fine-scale savanna heterogeneity for reptiles and small mammals. Biological Conservation. 2010; 143:2504–2513

[52] Woinarski JCZ, Legge S. The impacts of fire on birds in Australia's tropical savannas. Emu. 2013; 113:319–352.

[53] Whitehead PJ, Bowman DMJS, Tidemann SC. Biogeographic patterns, environmental correlates and conservation of avifauna in the Northern Territory, Australia. Journal of Biogeography. 1992; 19:151–161.

[54] Long RA, Donovan TM, Mackay P, Zielinski WJ, Buzas JS. Comparing scat detection dogs, cameras, and hair snares for surveying carnivores. Journal of Wildlife Management. 2007; 71:2018–2025.

[55] Balme GA, Hunter LTB, Slotow R. Evaluating Methods for Counting Cryptic Carnivores. Journal of Wildlife Management. 2009; 73:433–441.

[56] Brotons L, Wilfried T, Araújo M, Hirzel A. Presence‐absence versus presence‐only modelling methods for predicting bird habitat suitability. Ecography. 2004; 27:437–448.

[57] Rota C, Fletcher R, Evans J, Hutto R. Does accounting for imperfect detection improve species distribution models. Ecography. 2013; 34:659–670.

[58] Franklin J. Mapping species distributions: spatial inference and prediction. Cambridge; Cambridge University Press; 2010.

[59] Guillera-Arroita G. Modelling of species distributions, range dynamics and communities under imperfect detection: advances, challenges and opportunities. Ecography. 2017; 40:281–295.

[60] Austin MP. Spatial prediction of species distribution: an interface between ecological theory and statistical modelling. Ecological Modelling. 2002; 157:101–118.

[61] Legge S, Murphy BP, McGregor H, Woinarski JCZ, Augusteyn J, Ballard G, et al Enumerating a continental-scale threat: How many feral cats are in Australia? Biological Conservation. 2017; 206:293–303.

[62] Guillera-Arroita G, Lahoz-Monfort JJ. Designing studies to detect differences in species occupancy: power analysis under imperfect detection. Methods in Ecology and Evolution. 2012; 3:860–869.

[63] Lahoz-Monfort JJ, Guillera-Arroita G, Wintle BA. Imperfect detection impacts the performance of species distribution models. Global Ecology and Biogeography. 2014; 23:504–515.

[64] Kéry M. Extinction rate estimates for plant populations in revisitation studies: importance of detectability Conservation. Biology. 2004; 18:570–574.

