# Correction: Occupancy and detectability modelling of vertebrates in northern Australia using multiple sampling methods

**DOI:** 10.1371/journal.pone.0206373

**Published:** 2018-10-18

**Authors:** Luke D. Einoder, Darren M. Southwell, José J. Lahoz-Monfort, Graeme R. Gillespie, Alaric Fisher, Brendan A. Wintle

In the Methods section, there is an error in the second equation in the section titled "Occupancy and detectability modelling." The use of the symbol for division is incorrect. It should instead be the symbol for “conditional upon.” Please view the complete, correct equation here:

$$y_{ij}|z_{i}∼Bernoulli\left(z_{i}\rho_{ij}\right)$$
