# Supplementary figures and images for: Occupancy and detectability modelling of vertebrates in northern Australia using multiple sampling methods
Source: PLoS One. 2018 Sep 24;13(9):e0203304. doi: 10.1371/journal.pone.0203304 (PMC6152866; doi:10.1371/journal.pone.0203304)

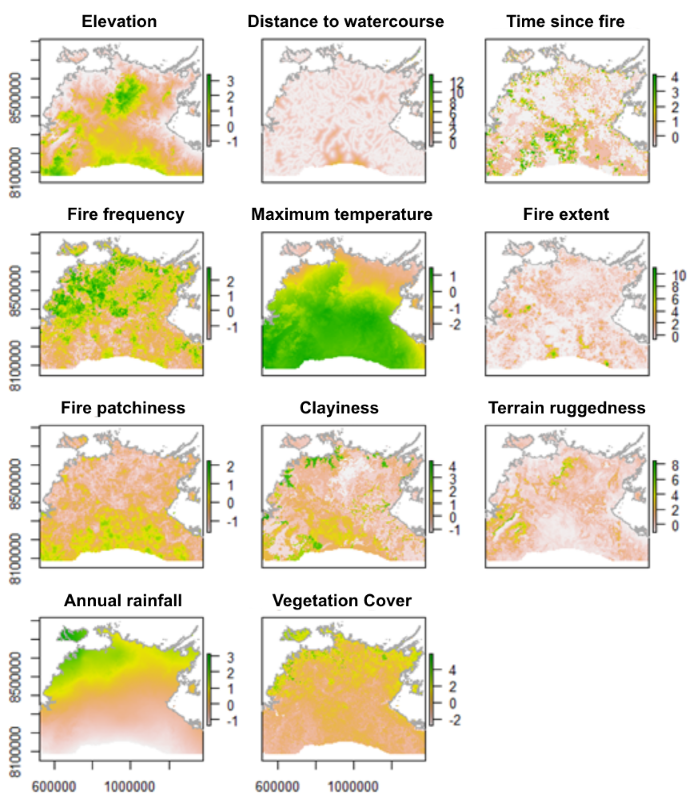

Supplement: S1 Fig — Covariates (scaled) at 1 km resolution used to model occupancy and detectability of 242 birds, mammals and reptiles recorded at 333 sites across the Top End of northern Australia. (TIF) [file pone.0203304.s001.tif]

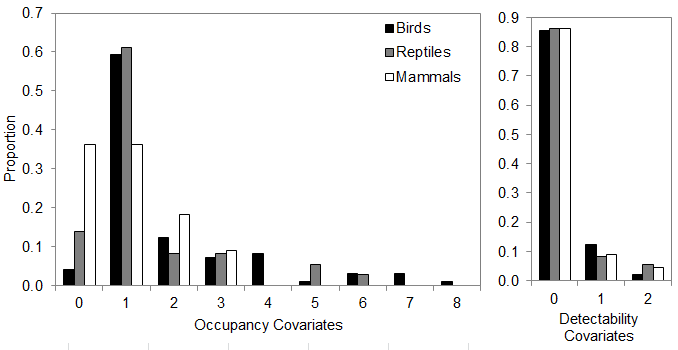

Supplement: S2 Fig — Proportion of models per animal group with 1–8 covariates included in the best model. Note, maximum covariate count for detectability models is 3, and method was included in detectability models for species with multiple methods of detection, but was not included in the covariate count presented here. (TIF) [file pone.0203304.s002.tif]

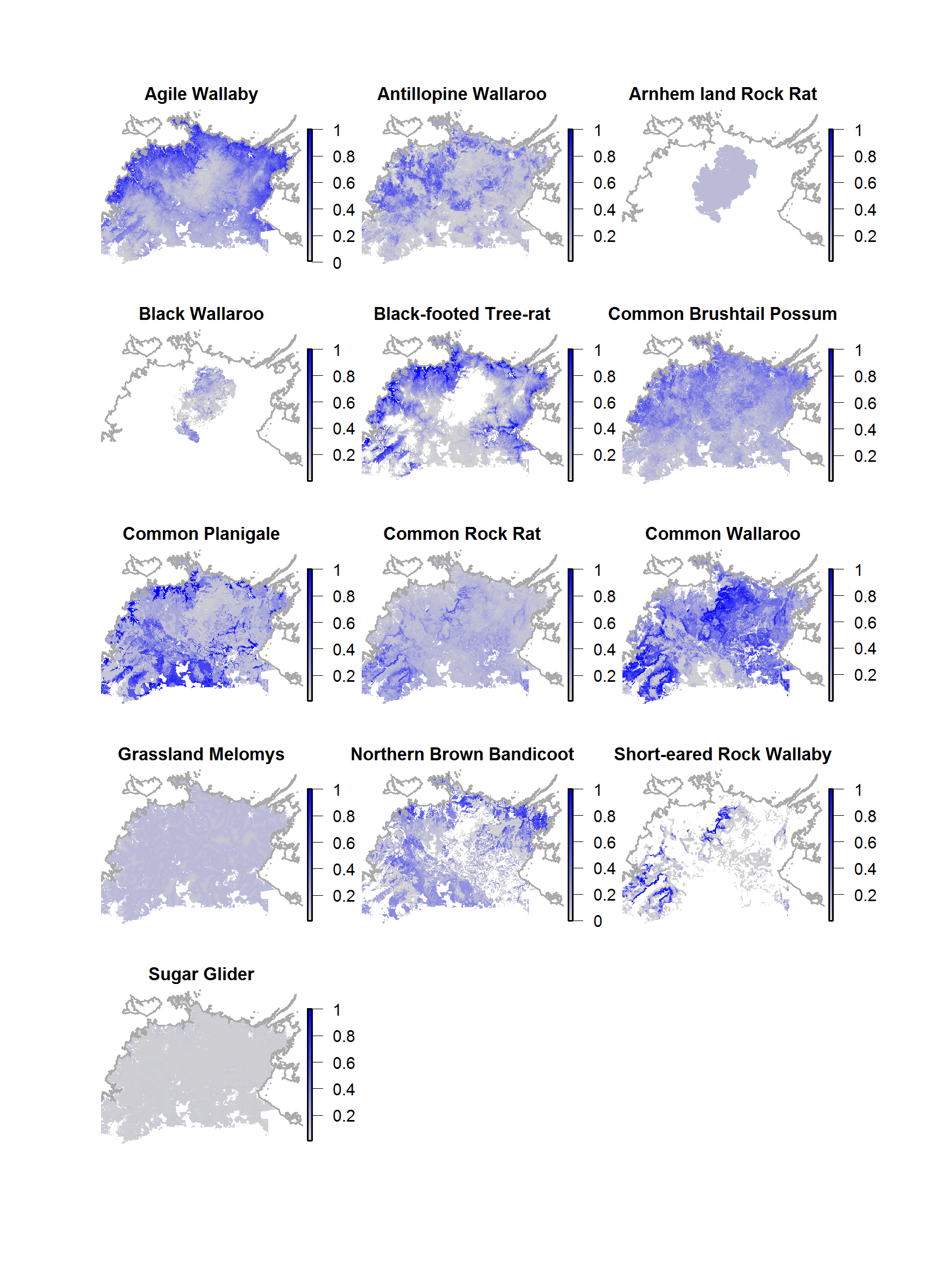

Supplement: S3 Fig — Occupancy maps for mammals with covariates in the best model. Light grey represents zero occupancy, while blue represents an occupancy probability of 1. (TIFF) [file pone.0203304.s003.tiff]

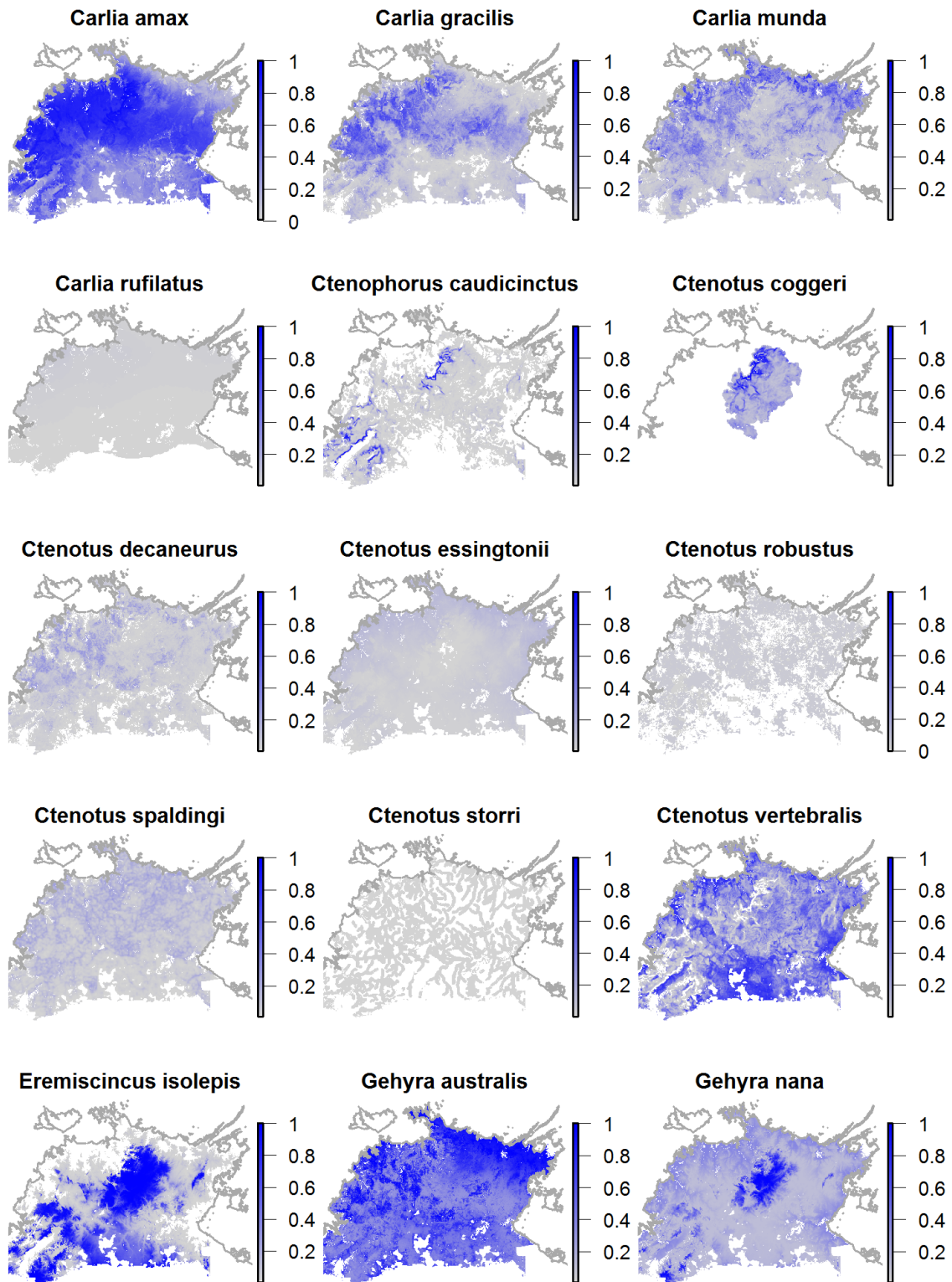

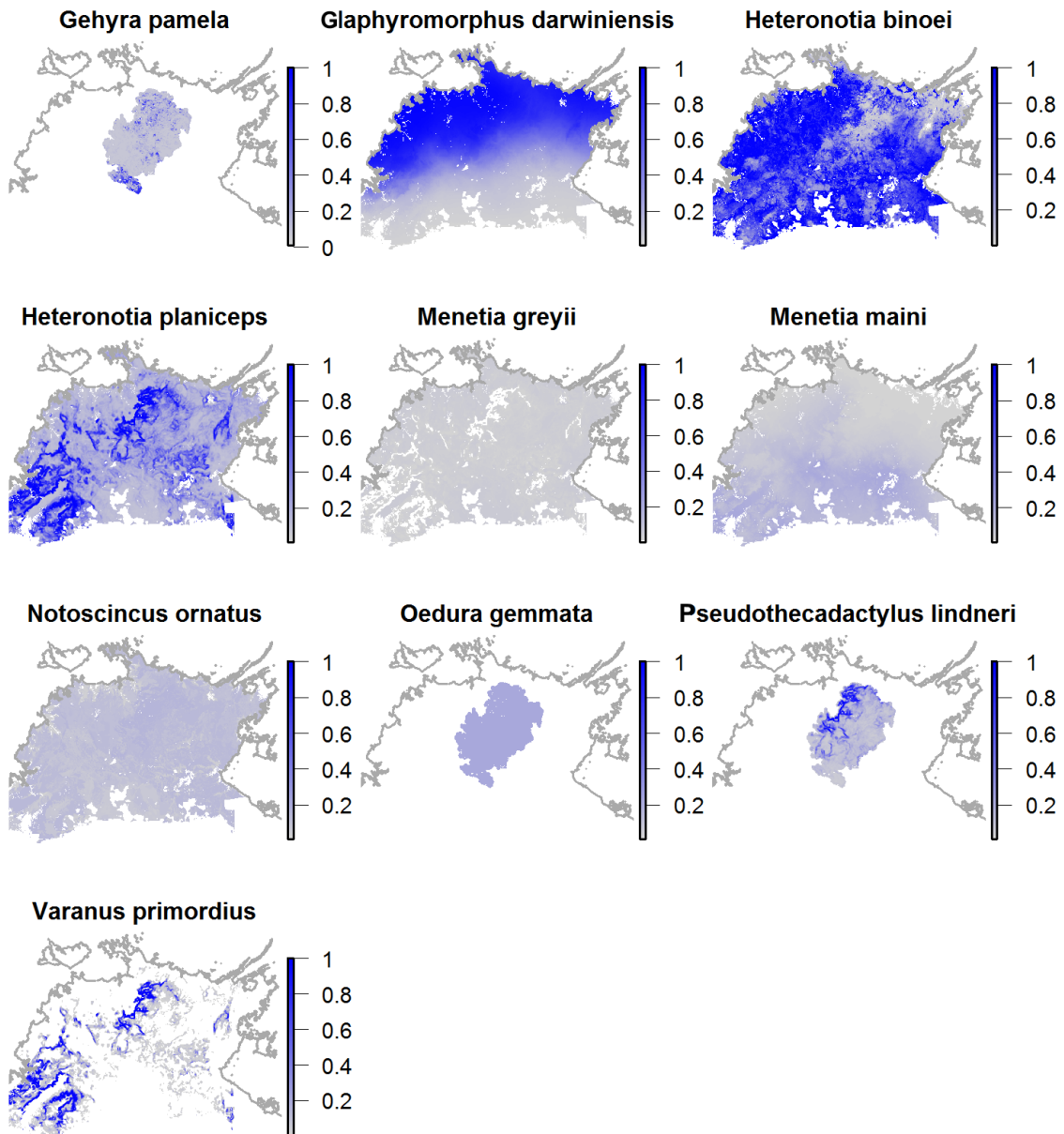

Supplement: S4 Fig — Occupancy maps for reptiles with covariates in the best model. Occupancy maps for reptiles with covariates in the best model. Light grey represents zero occupancy, while blue represents an occupancy probability of 1. (PDF) [file pone.0203304.s004.pdf]

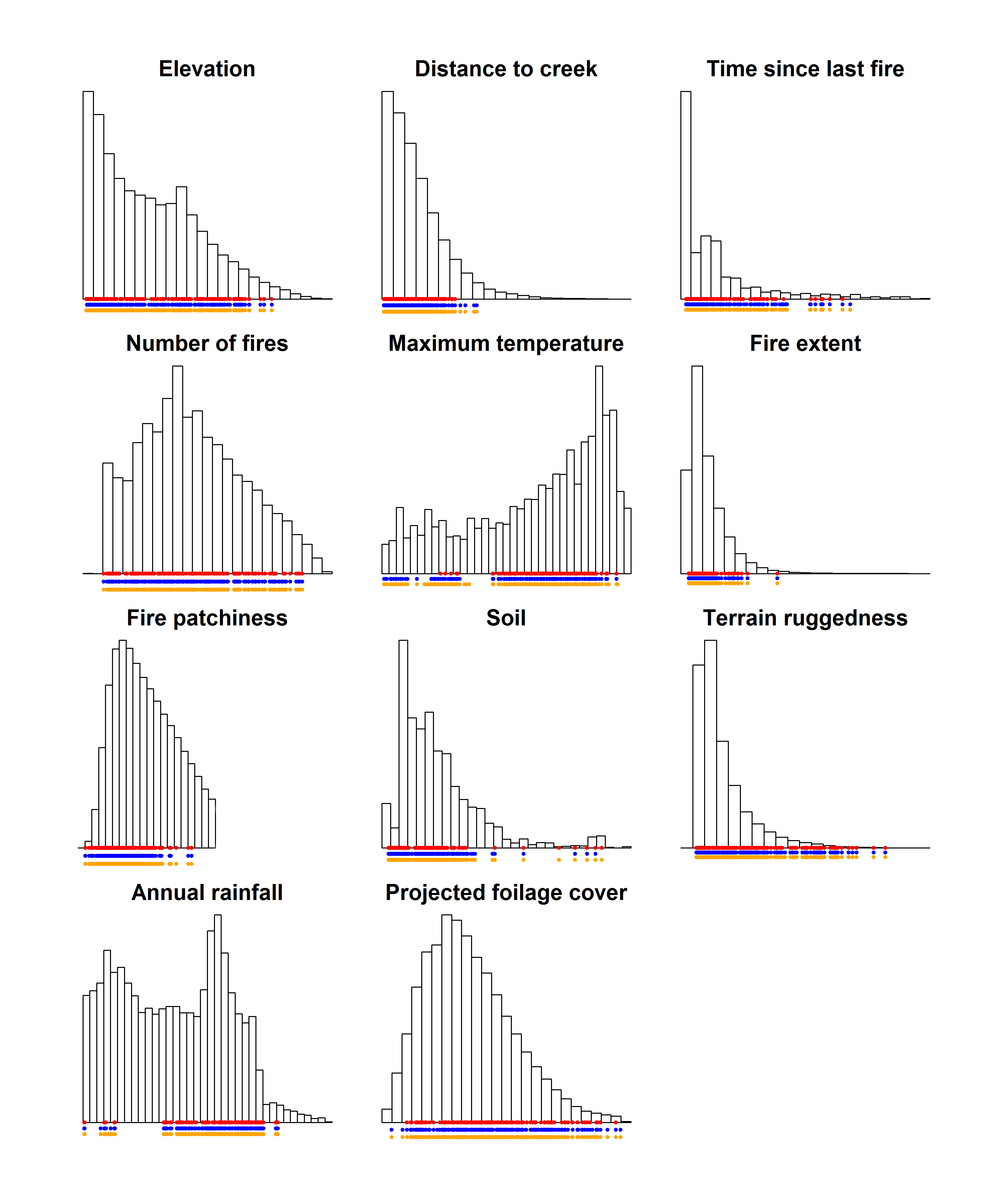

Supplement: S6 Fig — Frequency histograms of covariate values occurring within the truncated (i.e., not the full extent of the Top End) mapping region, showing the representativeness of sampling sites for birds (red), mammals (blue), and reptiles (orange) in comparison to the spectrum of environmental conditions to which species occupancy was predicted. (TIFF) [file pone.0203304.s006.tiff]
