# Supplementary material for: Occupancy and detectability modelling of vertebrates in northern Australia using multiple sampling methods
Source: PLoS One. 2018 Sep 24;13(9):e0203304. doi: 10.1371/journal.pone.0203304 (PMC6152866; doi:10.1371/journal.pone.0203304)
Supplement: S5 Fig — Occupancy maps for birds with covariates in the best model. Occupancy maps for birds with covariates in the best model. Light grey represents zero occupancy, while blue represents an occupancy probability of 1. (PDF) [file pone.0203304.s005.pdf]

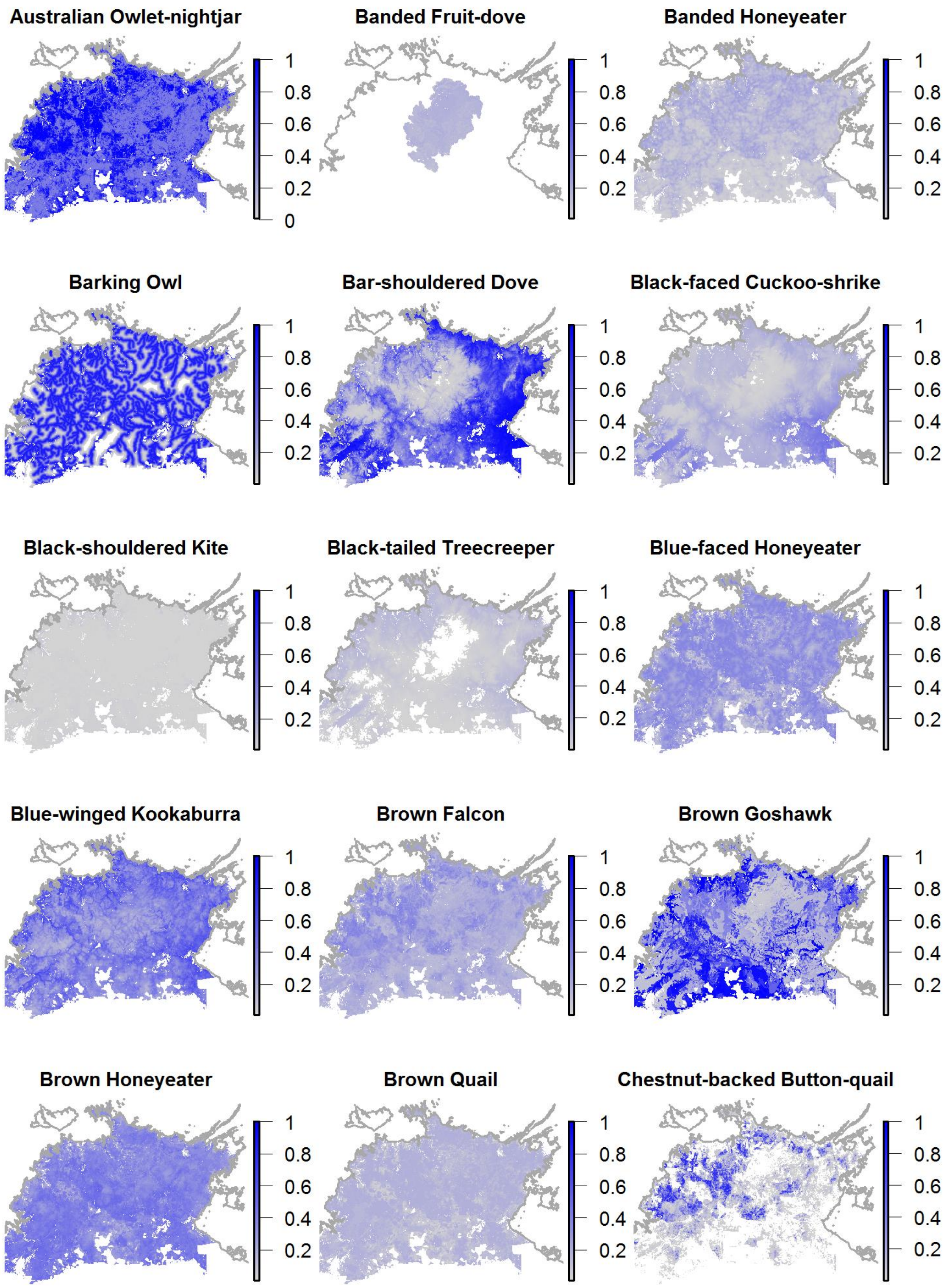
Australian Owlet-nightjar
1 0.8 0.6 0.4 0.2 0
Banded Fruit-dove
1 0.8 0.6 0.4 0.2
Banded Honeyeater
1 0.8 0.6 0.4 0.2
Barking Owl
1 0.8 0.6 0.4 0.2
Bar-shouldered Dove
1 0.8 0.6 0.4 0.2
Black-faced Cuckoo-shrike
1 0.8 0.6 0.4 0.2
Black-shouldered Kite
1 0.8 0.6 0.4 0.2
Black-tailed Treecreeper
1 0.8 0.6 0.4 0.2
Blue-faced Honeyeater
1 0.8 0.6 0.4 0.2
Blue-winged Kookaburra
1 0.8 0.6 0.4 0.2
Brown Falcon
1 0.8 0.6 0.4 0.2
Brown Goshawk
1 0.8 0.6 0.4 0.2
Brown Honeyeater
1 0.8 0.6 0.4 0.2
Brown Quail
1 0.8 0.6 0.4 0.2
Chestnut-backed Button-quail
1 0.8 0.6 0.4 0.2

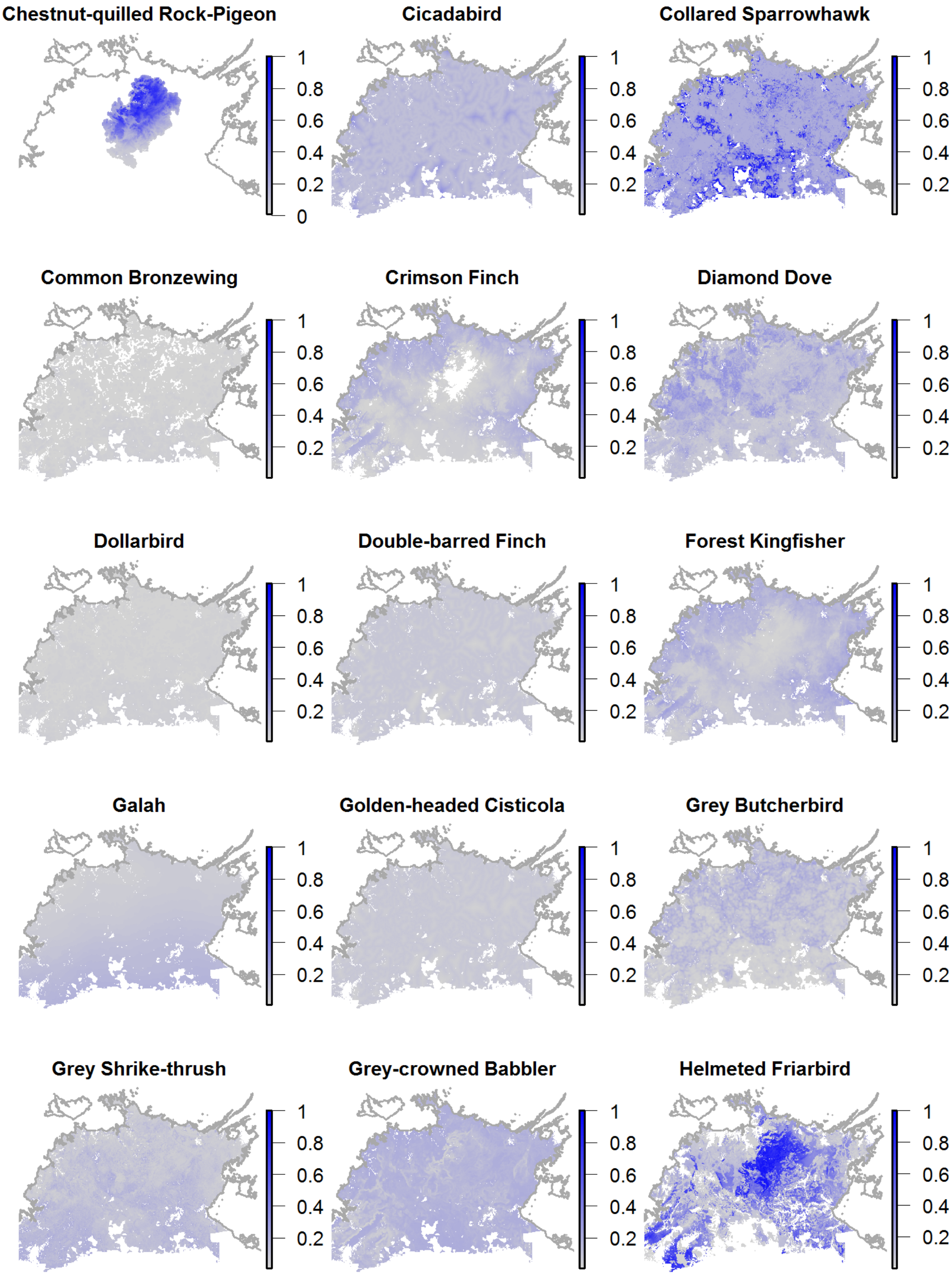
Chestnut-quilled Rock-Pigeon
1
0.8
0.6
0.4
0.2
0
Cicadabird
1
0.8
0.6
0.4
0.2
Collared Sparrowhawk
1
0.8
0.6
0.4
0.2
Common Bronzewing
1
0.8
0.6
0.4
0.2
Crimson Finch
1
0.8
0.6
0.4
0.2
Diamond Dove
1
0.8
0.6
0.4
0.2
Dollarbird
1
0.8
0.6
0.4
0.2
Double-barred Finch
1
0.8
0.6
0.4
0.2
Forest Kingfisher
1
0.8
0.6
0.4
0.2
Galah
1
0.8
0.6
0.4
0.2
Golden-headed Cisticola
1
0.8
0.6
0.4
0.2
Grey Butcherbird
1
0.8
0.6
0.4
0.2
Grey Shrike-thrush
1
0.8
0.6
0.4
0.2
Grey-crowned Babbler
1
0.8
0.6
0.4
0.2
Helmeted Friarbird
1
0.8
0.6
0.4
0.2

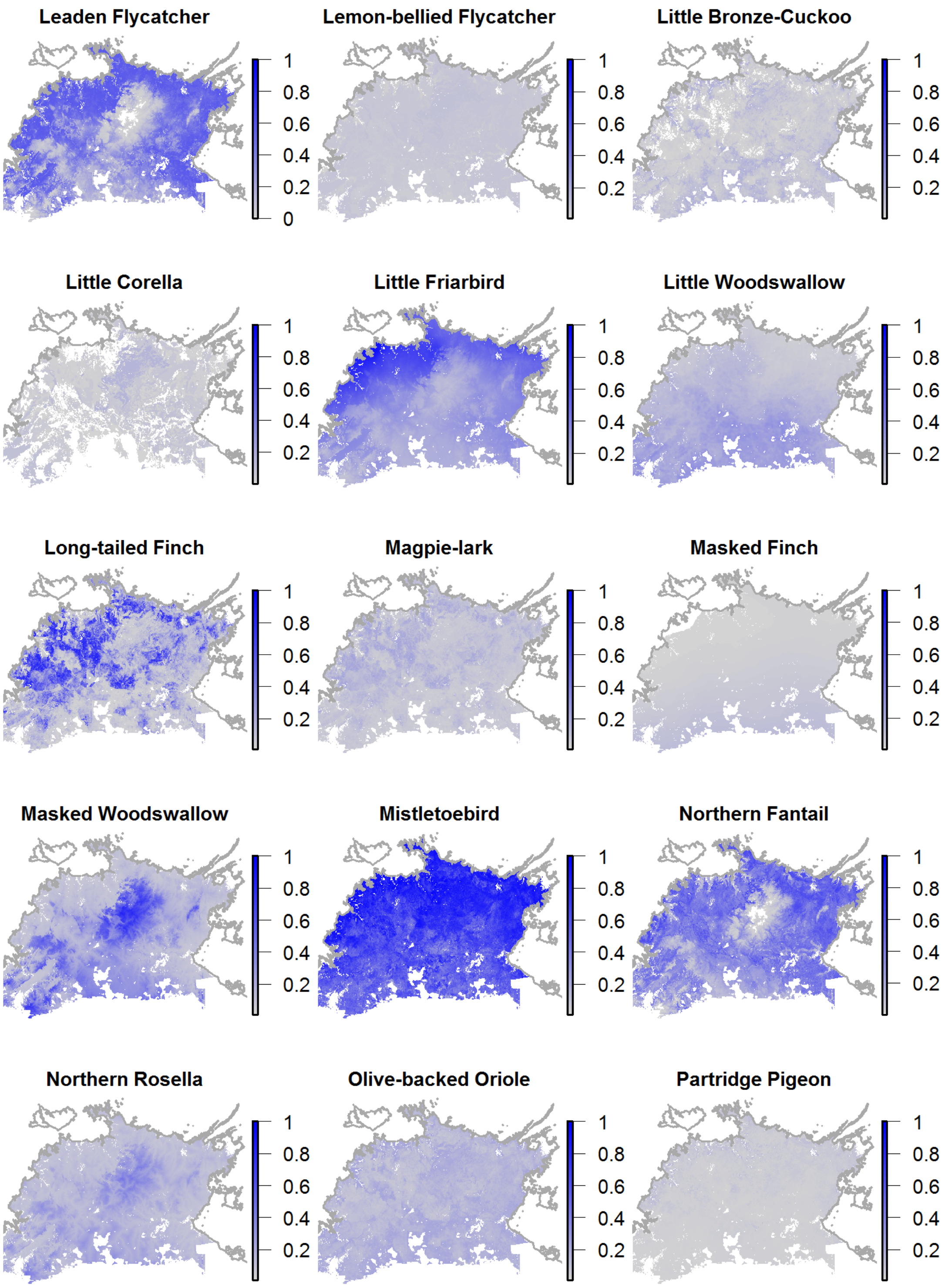
Leaden Flycatcher
1
0.8
0.6
0.4
0.2
0
Lemon-bellied Flycatcher
1
0.8
0.6
0.4
0.2
Little Bronze-Cuckoo
1
0.8
0.6
0.4
0.2
Little Corella
1
0.8
0.6
0.4
0.2
Little Friarbird
1
0.8
0.6
0.4
0.2
Little Woodswallow
1
0.8
0.6
0.4
0.2
Long-tailed Finch
1
0.8
0.6
0.4
0.2
Magpie-lark
1
0.8
0.6
0.4
0.2
Masked Finch
1
0.8
0.6
0.4
0.2
Masked Woodswallow
1
0.8
0.6
0.4
0.2
Mistletoebird
1
0.8
0.6
0.4
0.2
Northern Fantail
1
0.8
0.6
0.4
0.2
Northern Rosella
1
0.8
0.6
0.4
0.2
Olive-backed Oriole
1
0.8
0.6
0.4
0.2
Partridge Pigeon
1
0.8
0.6
0.4
0.2

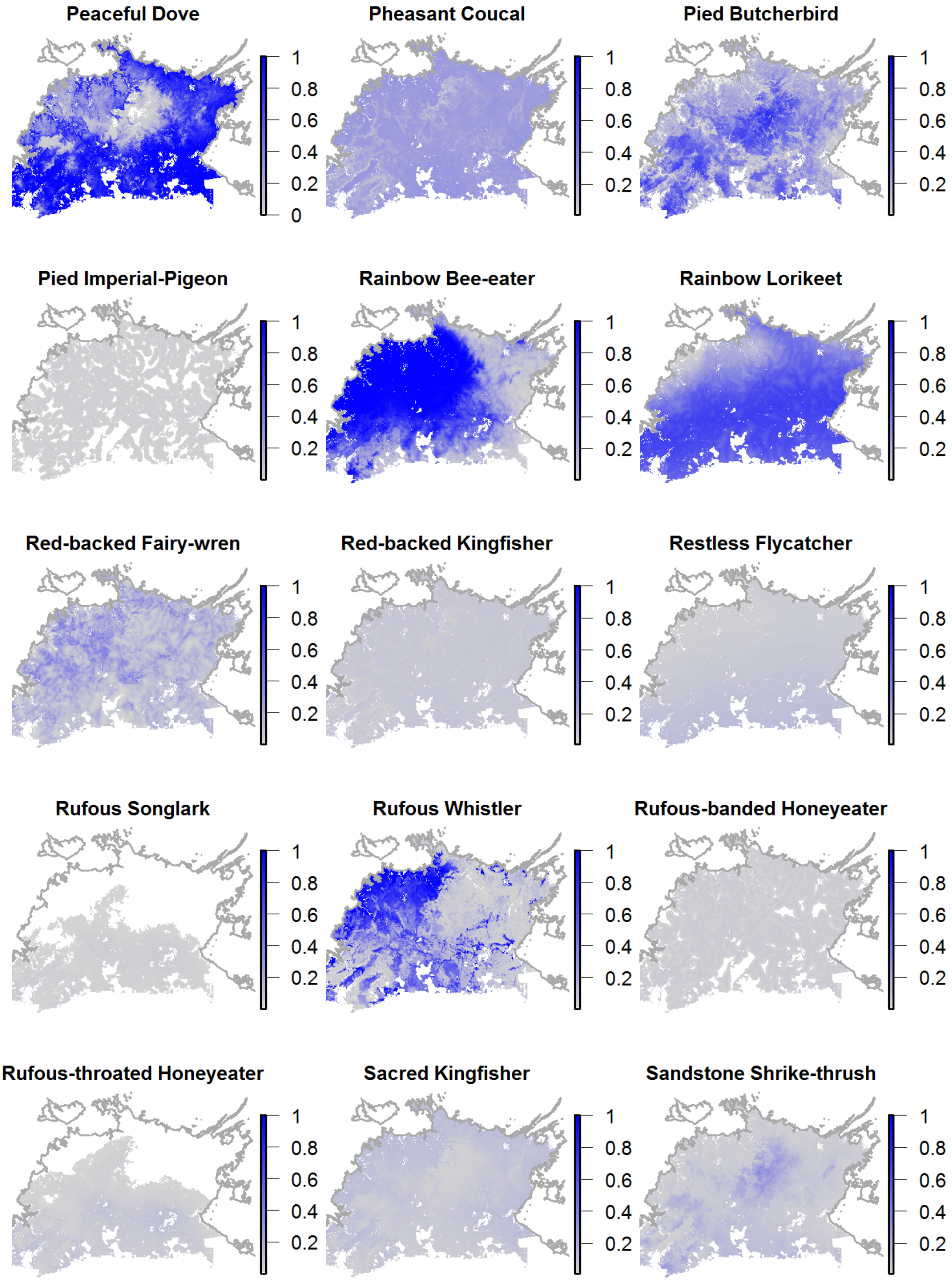
Peaceful Dove
1 0.8 0.6 0.4 0.2 0
Pheasant Coucal
1 0.8 0.6 0.4 0.2
Pied Butcherbird
1 0.8 0.6 0.4 0.2
Pied Imperial-Pigeon
1 0.8 0.6 0.4 0.2
Rainbow Bee-eater
1 0.8 0.6 0.4 0.2
Rainbow Lorikeet
1 0.8 0.6 0.4 0.2
Red-backed Fairy-wren
1 0.8 0.6 0.4 0.2
Red-backed Kingfisher
1 0.8 0.6 0.4 0.2
Restless Flycatcher
1 0.8 0.6 0.4 0.2
Rufous Songlark
1 0.8 0.6 0.4 0.2
Rufous Whistler
1 0.8 0.6 0.4 0.2
Rufous-banded Honeyeater
1 0.8 0.6 0.4 0.2
Rufous-throated Honeyeater
1 0.8 0.6 0.4 0.2
Sacred Kingfisher
1 0.8 0.6 0.4 0.2
Sandstone Shrike-thrush
1 0.8 0.6 0.4 0.2

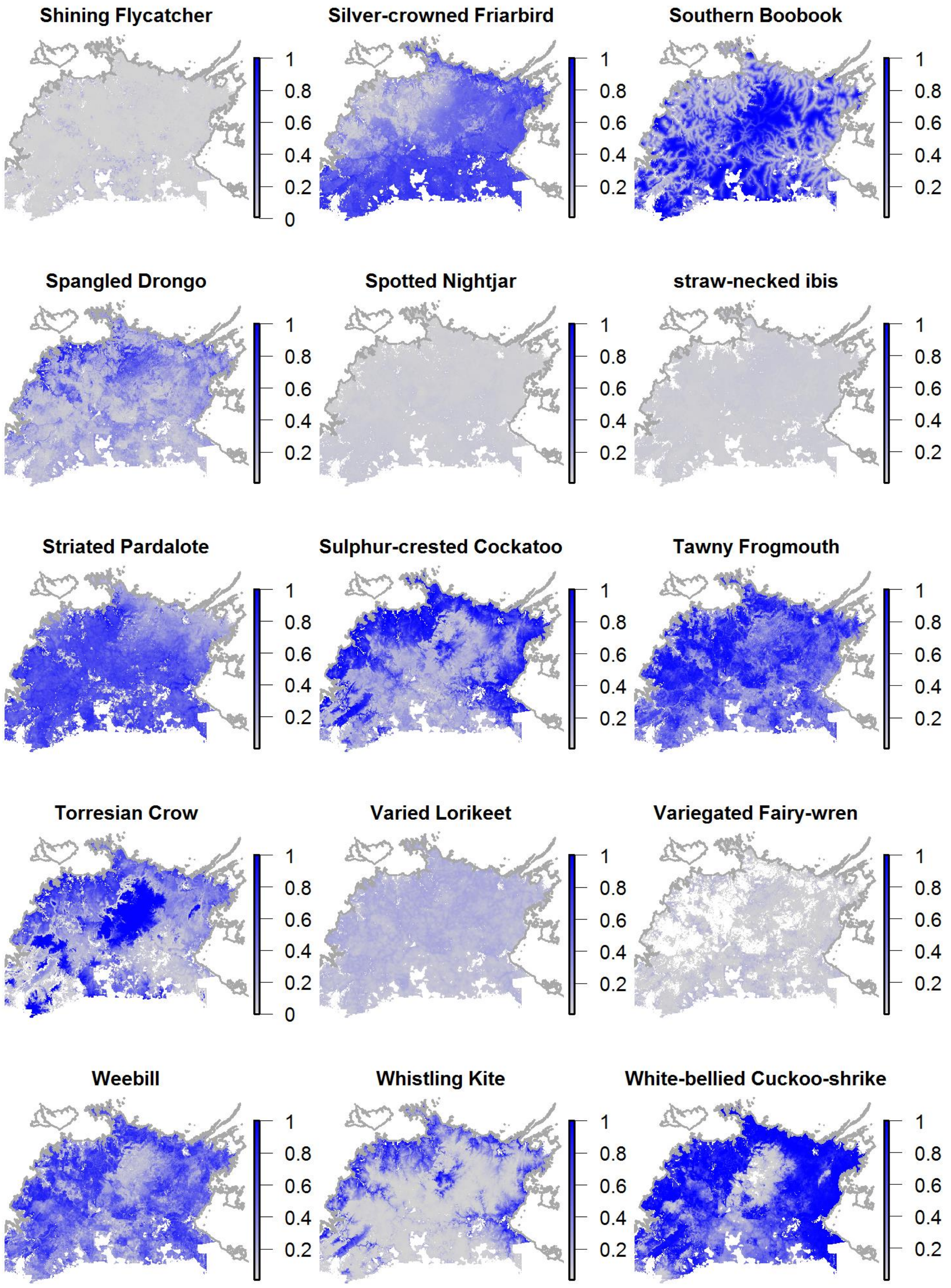

Shining Flycatcher
Silver-crowned Friarbird
Southern Boobook
Spangled Drongo
Spotted Nightjar
straw-necked ibis
Striated Pardalote
Sulphur-crested Cockatoo
Tawny Frogmouth
Torresian Crow
Varied Lorikeet
Variegated Fairy-wren
Weebill
Whistling Kite
White-bellied Cuckoo-shrike

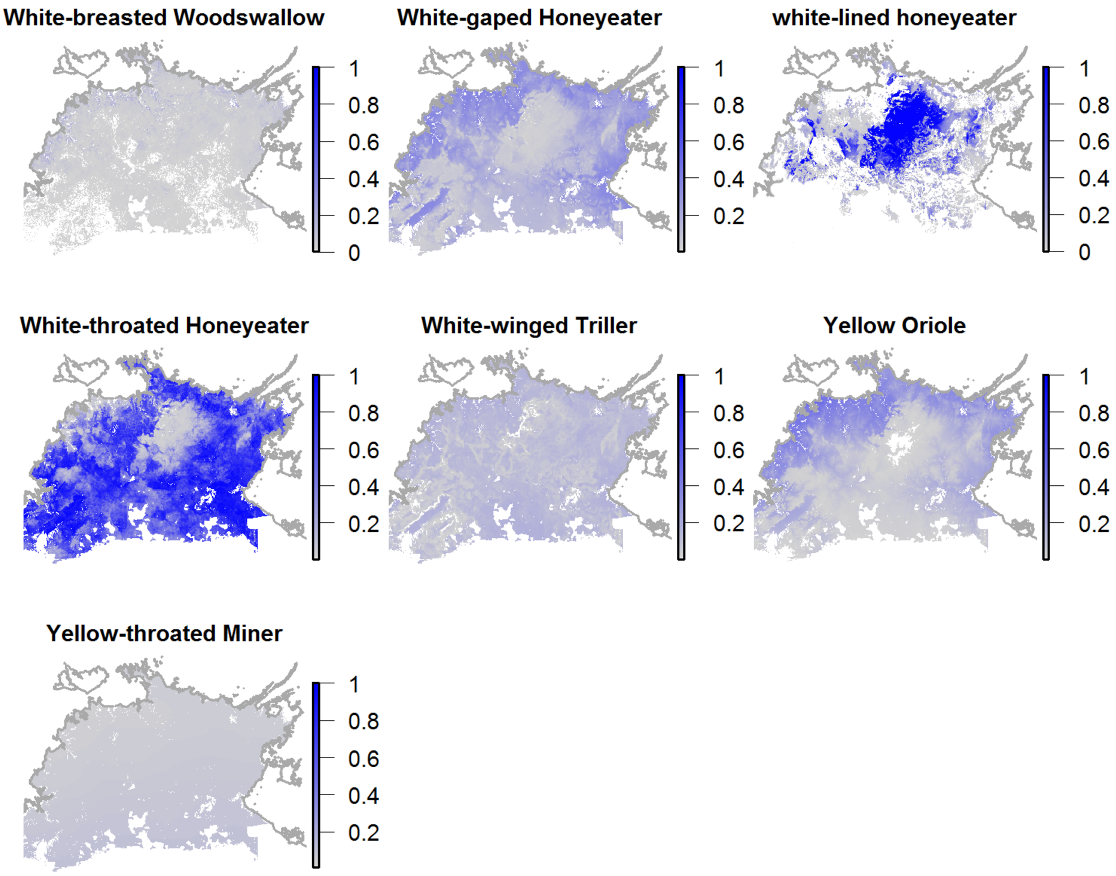
White-breasted Woodswallow
1
0.8
0.6
0.4
0.2
0
White-gaped Honeyeater
1
0.8
0.6
0.4
0.2
white-lined honeyeater
1
0.8
0.6
0.4
0.2
0
White-throated Honeyeater
1
0.8
0.6
0.4
0.2
White-winged Triller
1
0.8
0.6
0.4
0.2
Yellow Oriole
1
0.8
0.6
0.4
0.2
Yellow-throated Miner
1
0.8
0.6
0.4
0.2
