# Supplementary material for: Occupancy and detectability modelling of vertebrates in northern Australia using multiple sampling methods
Source: PLoS One. 2018 Sep 24;13(9):e0203304. doi: 10.1371/journal.pone.0203304 (PMC6152866; doi:10.1371/journal.pone.0203304)
Supplement: S1 Table — Summary of the method of detection pooled to generate detection histories per animal group per location: Djelk Indigenous Protected Area (DIPA); Fish River Station (FRS); Garig Gunak Barlu National Park (GGBNP); Gregory National Park (GNP); Kakadu National Park (KNP); Litchfield National Park (LNP); Nitmiluk National Park (NNP); Warrdeken Indigenous Protected Area (WIPA). Note, species-specific detection histories were generated from a subset of all methods available per animal group. (PDF) [file pone.0203304.s007.pdf]

Method	Mammal	Bird	Reptile	DIPA	FRS	GGBNP	GNP	KNP	LNP	NNP	WIPA	Total
Pitfall trap	X		X	20	19	24	11	145	32	43	28	322
Cage trap	X			20	19	24	11	145	32	43	28	322
Elliott trap	X			20	19	24	11	145	32	43	28	322
Camera trap	X			20	19	24	15	19	0	38	28	163
Nocturnal survey	X	X	X	0	0	0	11	145	31	41	0	228
Diurnal survey		X		0	0	0	6	145	31	43	0	225
