# Supplementary material for: Occupancy and detectability modelling of vertebrates in northern Australia using multiple sampling methods
Source: PLoS One. 2018 Sep 24;13(9):e0203304. doi: 10.1371/journal.pone.0203304 (PMC6152866; doi:10.1371/journal.pone.0203304)
Supplement: S2 Table — Covariates with a Spearman’s correlation coefficient greater than 0.7 are shown in bold, with one of a pair excluded from the analysis. (PDF) [file pone.0203304.s008.pdf]

| | Elevation | Maximum temperature | Minimum temperature | Annual rainfall | Veg. cover | Terrain ruggedness | Distance to watercourse | Clay content | Time since fire | Fire Frequency | Proportion burnt | Fire extent | Fire patchiness |
|---|---|---|---|---|---|---|---|---|---|---|---|---|---|
| Elevation | 1.00 | -0.01 | **-0.74** | -0.57 | 0.00 | 0.00 | 0.27 | 0.01 | -0.09 | -0.06 | -0.20 | 0.28 | 0.14 |
| Maximum temperature | -0.01 | 1.00 | -0.39 | -0.68 | 0.00 | 0.01 | -0.20 | -0.01 | -0.28 | 0.19 | 0.15 | 0.29 | -0.33 |
| Minimum temperature | **-0.74** | -0.39 | 1.00 | **0.86** | 0.00 | 0.00 | 0.07 | 0.00 | 0.38 | -0.29 | -0.14 | -0.49 | 0.18 |
| Annual rainfall | -0.57 | -0.68 | **0.86** | 1.00 | 0.00 | -0.01 | 0.09 | 0.00 | 0.27 | -0.15 | -0.04 | -0.40 | 0.15 |
| Vegetation cover | 0.00 | 0.00 | 0.00 | 0.00 | 1.00 | -0.04 | 0.01 | 0.18 | 0.00 | 0.00 | 0.00 | 0.00 | 0.00 |
| Terrain ruggedness | 0.00 | 0.01 | 0.00 | -0.01 | -0.04 | 1.00 | -0.01 | -0.40 | 0.00 | 0.00 | 0.00 | 0.01 | 0.00 |
| Distance to creek | 0.27 | -0.20 | 0.07 | 0.09 | 0.01 | -0.01 | 1.00 | -0.01 | 0.05 | -0.11 | -0.13 | 0.02 | 0.29 |
| Clay content | 0.01 | -0.01 | 0.00 | 0.00 | 0.18 | -0.40 | -0.01 | 1.00 | -0.01 | 0.00 | 0.00 | -0.01 | -0.01 |
| Time since fire | -0.09 | -0.28 | 0.38 | 0.27 | 0.00 | 0.00 | 0.05 | -0.01 | 1.00 | -0.70 | -0.58 | -0.44 | 0.50 |
| Fire Frequency | -0.06 | 0.19 | -0.29 | -0.15 | 0.00 | 0.00 | -0.11 | 0.00 | -0.70 | 1.00 | **0.76** | 0.57 | -0.46 |
| Proportion burnt | -0.20 | 0.15 | -0.14 | -0.04 | 0.00 | 0.00 | -0.13 | 0.00 | -0.58 | **0.76** | 1.00 | 0.66 | -0.58 |
| Fire extent | 0.28 | 0.29 | -0.49 | -0.40 | 0.00 | 0.01 | 0.02 | -0.01 | -0.44 | 0.57 | 0.66 | 1.00 | -0.23 |
| Fire patchiness | 0.14 | -0.33 | 0.18 | 0.15 | 0.00 | 0.00 | 0.29 | -0.01 | 0.50 | -0.46 | -0.58 | -0.23 | 1.00 |
