# Supplementary material for: Occupancy and detectability modelling of vertebrates in northern Australia using multiple sampling methods
Source: PLoS One. 2018 Sep 24;13(9):e0203304. doi: 10.1371/journal.pone.0203304 (PMC6152866; doi:10.1371/journal.pone.0203304)
Supplement: S3 Table — Model coefficients for the occupancy component of the reptile models. Note, species containing only dashes were recorded during surveys but were unable to be modelled. (PDF) [file pone.0203304.s009.pdf]

Species	Int	Clay	Clay^2	Veg. cover	Veg. cover^2	Elev.	Elev.^{2}	Dist. to water.	Terr. rugg.	Max. temp.	Max. temp.^{2}	Ann. rain	Ann. rain^2	Fire freq.	Fire freq.^{2}	Time since fire	Time since fire^2	Fire extent	Fire patch.
Carlia amax	0.7	-0.35	-	-	-	-	-	-	0.19	-0.34	-0.89	0.79	-	-	-	-	-	-	-
Carlia gracilis	-1.15	-	-	-	-	-	-	-	-	-0.98	-1.16	-	-	0.68	-	-	-	-	-
Carlia munda	-1.9	0.73	-0.12	0.04	-	-	-	-0.55	-	-0.52	-	-	-	0.67	-	-	-	-	-
Carlia rufilatus	-4.12	-	-	-	-	-	-	-	-	-	-	0.65	-	-	-	-	-	-	-
Carlia triacantha	-	-	-	-	-	-	-	-	-	-	-	-	-	-	-	-	-	-	-
Chlamydosaurus kingii	-	-	-	-	-	-	-	-	-	-	-	-	-	-	-	-	-	-	-
Cryptoblepharus sp.	-	-	-	-	-	-	-	-	-	-	-	-	-	-	-	-	-	-	-
Ctenophorus caudicinctus	-4.17	-	-	-	-	-	-	-	1.02	-	-	-	-	-	-	-	-	-	-
Ctenotus arnhemensis	-	-	-	-	-	-	-	-	-	-	-	-	-	-	-	-	-	-	-
Ctenotus borealis	-	-	-	-	-	-	-	-	-	-	-	-	-	-	-	-	-	-	-
Ctenotus coggeri	-0.84	-	-	-	-	-	-	-	0.74	-	-	-	-	-	-	-	-	-	0.85
Ctenotus decaneurus	-3.32	-	-	-	-	-	-	-	-	-	-	-	-	0.92	-	-	-	-	-
Ctenotus essingtonii	-3.06	-	-	0.05	-	-0.63	-	-	-	-0.2	-	-	-	-	-	-	-	-	-
Ctenotus inornatus	4.23	-	-	-0.25	-	-	-	-	-	-	-	-	-	-	-	-	-	-	-
Ctenotus pantherinus	0.85	-	-	-0.22	-	-	-	-	-	-	-	-	-	-	-	-	-	-	-
Ctenotus piankai	-5.59	-	-	-	-	-	-	-	-	2.49	-	-	-	-	-	-	-	-	-
Ctenotus quirinus	-	-	-	-	-	-	-	-	-	-	-	-	-	-	-	-	-	-	-
Ctenotus robustus	-5.38	-	-	-	-	-	-	-	-	-	-	-	-	-	-	-3.55	-	-	-
Ctenotus saxatilis	-	-	-	-	-	-	-	-	-	-	-	-	-	-	-	-	-	-	-
Ctenotus spaldingi	-3.9	-	-	-	-	-	-	-	-	-	-	-	-	-	-	-	-	-	-1.81
Ctenotus storri	-4.79	-	-	-	-	-	-	-0.91	-	-	-	-	-	-	-	-	-	-	-
Ctenotus vertebralis	-0.68	-	-	-	-	-	-	-	-1.27	-	-	-	-	-0.55	-	-	-	-	-
Delma borea	-	-	-	-	-	-	-	-	-	-	-	-	-	-	-	-	-	-	-
Delma tincta	-	-	-	-	-	-	-	-	-	-	-	-	-	-	-	-	-	-	-
Diplodactylus bilineata	-	-	-	-	-	-	-	-	-	-	-	-	-	-	-	-	-	-	-
Diplodactylus conspicillatus	-	-	-	-	-	-	-	-	-	-	-	-	-	-	-	-	-	-	-
Diporiphora albilabris	-	-	-	-	-	-	-	-	-	-	-	-	-	-	-	-	-	-	-
Diporiphora bennettii	-	-	-	-	-	-	-	-	-	-	-	-	-	-	-	-	-	-	-
Diporiphora bilineata	-	-	-	-	-	-	-	-	-	-	-	-	-	-	-	-	-	-	-
Diporiphora magna	-	-	-	-	-	-	-	-	-	-	-	-	-	-	-	-	-	-	-
Eremiscincus isolepis	-1.47	-	-	-	-	2.99	-	-	-	-	-	-	-	-	-	-	-	-	-
Gehyra australis	-0.85	-	-	-	-	-	-	-	-	0.73	-	-	-	0.58	-	-	-	-	-

Species	Int	Clay	Clay^2	Veg. cover	Veg. cover^2	Elev.	Elev.^{2}	Dist. to water.	Terr. rugg.	Max. temp.	Max. temp.^{2}	Ann. rain	Ann. rain^2	Fire freq.	Fire freq.^{2}	Time since fire	Time since fire^2	Fire extent	Fire patch.
Gehyra nana	-2.06	-	-	-	-	0.83	-	-	-	-	-	-	-	-	-	-	-	-	-
Gehyra pamela	-1.74	-	-	-	-	-	-	-	1.07	-	-	-	-	-	-	-	-	-	-
Glaphyromorphus darwiniensis	-1.02	-	-	-	-	-	-	-	-	-	-	3.04	-	-	-	-	-	-	-
Glaphyromorphus douglasi	-	-	-	-	-	-	-	-	-	-	-	-	-	-	-	-	-	-	-
Hemidactylus frenatus	-	-	-	-	-	-	-	-	-	-	-	-	-	-	-	-	-	-	-
Heteronotia binoei	0.55	1.47	-0.91	-	-	-	-	-	1.03	-0.98	-	-	-	0.93	-	-	-	-	-
Heteronotia planiceps	-1.07	-	-	-	-	-	-	-	1.3	-	-	-	-	-	-	-	-	-	-
Lerista karlschmidti	-	-	-	-	-	-	-	-	-	-	-	-	-	-	-	-	-	-	-
Lerista orientalis	-3.68	-	-	-	-	-	-	-	-	-	-	-	-	-	-	0.27	-	-	-
Lialis burtonis	-	-	-	-	-	-	-	-	-	-	-	-	-	-	-	-	-	-	-
Lophognathus gilberti	-	-	-	-	-	-	-	-	-	-	-	-	-	-	-	-	-	-	-
Lophognathus temporalis	-	-	-	-	-	-	-	-	-	-	-	-	-	-	-	-	-	-	-
Lucasium stenodactylum	-	-	-	-	-	-	-	-	-	-	-	-	-	-	-	-	-	-	-
Menetia alanae	-	-	-	-	-	-	-	-	-	-	-	-	-	-	-	-	-	-	-
Menetia concinna	-	-	-	-	-	-	-	-	-	-	-	-	-	-	-	-	-	-	-
Menetia greyii	-3.28	0.35	-	-	-	-	-	-	-	-	-	-	-	-	-	-	-	-	-
Menetia maini	-2.49	-	-	-	-	-	-	-	-	1.1	-	-	-	-	-	-	-	-	-
Morethia ruficauda	-1.83	-	-	-	-	-	-	-	-	-	-	-	-	-	-	-	-	-	-
Morethia storri	-2.47	-	-	-	-	-	-	-	-	-	-	-	-	-	-	-	-	-	-
Nephrurus sheai	-	-	-	-	-	-	-	-	-	-	-	-	-	-	-	-	-	-	-
Notoscincus ornatus	-2.26	-0.43	-	-	-	-	-	-	-	-	-	-	-	-	-	-	-	-	-
Oedura gemmata	-1.32	-	-	-	-	-	-	-	-	-	-	-	-	-	-	-	-	-	-
Oedura marmorata	-3.53	-	-	-	-	-	-	-	-	-	-	-	-	-	-	-	-	-	-
Oedura rhombifer	-	-	-	-	-	-	-	-	-	-	-	-	-	-	-	-	-	-	-
Proablepharus tenuis	-	-	-	-	-	-	-	-	-	-	-	-	-	-	-	-	-	-	-
Pseudonaja nuchalis	-	-	-	-	-	-	-	-	-	-	-	-	-	-	-	-	-	-	-
Pseudothecadactylus lindneri	-2.16	-	-	-	-	-	-	-	0.95	-	-	-	-	-	-	-	-	-	-
Ramphotyphlops sp.	-	-	-	-	-	-	-	-	-	-	-	-	-	-	-	-	-	-	-
Sphenomorphus sp.	-	-	-	-	-	-	-	-	-	-	-	-	-	-	-	-	-	-	-
Strophurus ciliaris	-	-	-	-	-	-	-	-	-	-	-	-	-	-	-	-	-	-	-
Strophurus taeniatus	-	-	-	-	-	-	-	-	-	-	-	-	-	-	-	-	-	-	-
Varanus acanthurus	-	-	-	-	-	-	-	-	-	-	-	-	-	-	-	-	-	-	-
Varanus baritji	-1.29	-	-	-	-	-	-	-	-	-	-	-	-	-	-	-	-	-	-
Varanus kingorum	-	-	-	-	-	-	-	-	-	-	-	-	-	-	-	-	-	-	-
Varanus primordius	-5.23	-	-	-	-	-	-	-	2.29	-	-	-	-	-	-	-	-	-	-
Varanus scalaris	-	-	-	-	-	-	-	-	-	-	-	-	-	-	-	-	-	-	-
Varanus tristis	-	-	-	-	-	-	-	-	-	-	-	-	-	-	-	-	-	-	-
