# Supplementary material for: Occupancy and detectability modelling of vertebrates in northern Australia using multiple sampling methods
Source: PLoS One. 2018 Sep 24;13(9):e0203304. doi: 10.1371/journal.pone.0203304 (PMC6152866; doi:10.1371/journal.pone.0203304)
Supplement: S4 Table — Note, species containing only dashes were recorded during surveys but were unable to be modelled. (PDF) [file pone.0203304.s010.pdf]

Species	Int	Clay	Clay^2	Veg. cover	Veg. cover^2	Elev.	Elev.^{2}	Dist. to water.	Terr. rugg.	Max. temp.	Max. temp.^{2}	Ann. rain	Ann. rain^2	Fire freq.	Fire freq.^{2}	Time since fire	Time since fire^2	Fire extent	Fire patch.
Apostlebird	-	-	-	-	-	-	-	-	-	-	-	-	-	-	-	-	-	-	-
Arafura Fantail	-3.88	-	-	-	-	-	-	-	-	-	-	-	-	-	-	-	-	-	-
Australasian Darter	-	-	-	-	-	-	-	-	-	-	-	-	-	-	-	-	-	-	-
Australasian Figbird	-	-	-	-	-	-	-	-	-	-	-	-	-	-	-	-	-	-	-
Australian Bustard	-	-	-	-	-	-	-	-	-	-	-	-	-	-	-	-	-	-	-
Australian Hobby	-	-	-	-	-	-	-	-	-	-	-	-	-	-	-	-	-	-	-
Australian Owlet-nightjar	-0.4	-	-	-	-	-	-	-	-	-	-	-	-	1.31	-	-	-	-	-
Australian Pelican	-	-	-	-	-	-	-	-	-	-	-	-	-	-	-	-	-	-	-
Australian White Ibis	-	-	-	-	-	-	-	-	-	-	-	-	-	-	-	-	-	-	-
Azure Kingfisher	-	-	-	-	-	-	-	-	-	-	-	-	-	-	-	-	-	-	-
Banded Fruit-dove	-2.02	-0.38	-	-	-	-	-	-	-	-	-	-	-	-	-	-	-	-	-
Banded Honeyeater	-3.16	-	-	-	-	-	-	-	-	-	-	-	-	-	-	-	-	-	-1.38
Bar-breasted Honeyeater	-	-	-	-	-	-	-	-	-	-	-	-	-	-	-	-	-	-	-
Barking Owl	-0.61	-	-	-	-	-	-	-3.6	-	-	-	-	-	-	-	-	-	-	-
Bar-shouldered Dove	0.33	-	-	-	-	-1.54	-	-	-	-1.45	-0.44	-0.63	-	-0.59	-	-0.39	-	-	-
Black Bittern	-	-	-	-	-	-	-	-	-	-	-	-	-	-	-	-	-	-	-
Black Kite	-	-	-	-	-	-	-	-	-	-	-	-	-	-	-	-	-	-	-
Black-breasted Buzzard	-	-	-	-	-	-	-	-	-	-	-	-	-	-	-	-	-	-	-
Black-chinned Honeyeater	-	-	-	-	-	-	-	-	-	-	-	-	-	-	-	-	-	-	-
Black-faced Cuckoo-shrike	-1.72	-	-	-	-	-0.79	-	-	-	-0.8	-	-	-	-	-	-	-	-	-
Black-faced Woodswallow	-	-	-	-	-	-	-	-	-	-	-	-	-	-	-	-	-	-	-
Black-shouldered Kite	-3.95	-	-	-	-	-	-	-	-	-	-	-	-	-	-	-	-	0.3	-
Black-tailed Treecreeper	-3.34	-	-	-	-	-1.12	-	-	-	-	-	-	-	-	-	-	-	-	-
Blue-faced Honeyeater	-1.19	-	-	-	-	-	-	-0.43	-	-	-	-	-	-	-	-0.55	-	-	-
Blue-winged Kookaburra	-0.74	-	-	-	-	-0.35	-	-	-	-	-	-	-	-0.41	-	-	-	-	-0.53
Broad-billed Flycatcher	-	-	-	-	-	-	-	-	-	-	-	-	-	-	-	-	-	-	-
Brown Falcon	-1.44	-	-	-	-	-	-	-	-	-	-	-	-	0.39	-	-	-	-	-
Brown Goshawk	0.22	-	-	-	-	-	-	-	-	-	-	-	-	-	-	-	-	2.54	-
Brown Honeyeater	-0.54	-	-	-0.26	-	-	-	-0.18	-	-	-	-	-	-	-	-0.27	-	-	-
Brown Quail	-1.97	-	-	-	-	-	-	-	-	-	-	-	-	-	-	-0.56	-	-	-
Brush Cuckoo	-	-	-	-	-	-	-	-	-	-	-	-	-	-	-	-	-	-	-
Bush Stone-curlew	-	-	-	-	-	-	-	-	-	-	-	-	-	-	-	-	-	-	-
Cattle Egret	-	-	-	-	-	-	-	-	-	-	-	-	-	-	-	-	-	-	-
Channel-billed Cuckoo	-	-	-	-	-	-	-	-	-	-	-	-	-	-	-	-	-	-	-
Chestnut-backed Button-quail	-4.5	-	-	-	-	-	-	-	-	-	-	-	-	2.29	-	-	-	-	-

Species	Int	Clay	Clay^2	Veg. cover	Veg. cover^2	Elev.	Elev.^{2}	Dist. to water.	Terrain rugg.	Max. temp.	Max. temp.^{2}	Ann. rain	Ann. rain^2	Fire freq.	Fire freq.^{2}	Time since fire	Time since fire^2	Fire extent	Fire patch.
Chestnut-breasted Mannikin	-	-	-	-	-	-	-	-	-	-	-	-	-	-	-	-	-	-	-
Chestnut-quilled Rock-Pigeon	-2.83	-	-	-	-	0.86	-	-	-	2.1	-	-	-	-	-	-	-	-	-
Cicadabird	-1.88	-	-	-	-	-	-	0.41	-	-	-	-	-	-	-	-	-	-	-
Collared Sparrowhawk	-0.89	-	-	-	-	-	-	-	-	-	-	-	-	-	-	0.87	-	-	-
Common Bronzewing	-3.42	-	-	-	-	-	-	-	-	-	-	-	-	-	-	-	-	-	0.93
Crimson Finch	-2.87	-	-	-	-	-1.02	-	-	-	-	-	-	-	-	-	-	-	-	-
Diamond Dove	-2.17	-	-	-	-	-	-	-	-	-	-	-	-	0.53	-	-	-	-	-
Dollarbird	-2.17	-	-	-	-	-	-	-	-	-	-	-	-	-	-	-	-	-	0.59
Double-barred Finch	-2.88	-	-	-	-	-	-	-0.55	-	-	-	-	-	-	-	-	-	-	-
Dusky Grasswren	-	-	-	-	-	-	-	-	-	-	-	-	-	-	-	-	-	-	-
Dusky Honeyeater	-	-	-	-	-	-	-	-	-	-	-	-	-	-	-	-	-	-	-
Eastern Great Egret	-	-	-	-	-	-	-	-	-	-	-	-	-	-	-	-	-	-	-
Eastern Koel	-	-	-	-	-	-	-	-	-	-	-	-	-	-	-	-	-	-	-
Emerald Dove	-	-	-	-	-	-	-	-	-	-	-	-	-	-	-	-	-	-	-
Forest Kingfisher	-2.05	-	-	-	-	-0.69	-	-	-	-	-	-	-	-	-	-	-	-	0.37
Fork-tailed Swift	-	-	-	-	-	-	-	-	-	-	-	-	-	-	-	-	-	-	-
Galah	-2.27	-	-	-	-	-	-	-	-	-0.68	-	-	-	-	-	-	-	-	-
Golden-headed Cisticola	-2.89	-	-	-	-	-	-	-0.41	-	-	-	-	-	-	-	-	-	-	-
Great Bowerbird	-	-	-	-	-	-	-	-	-	-	-	-	-	-	-	-	-	-	-
Green-backed Gerygone	-	-	-	-	-	-	-	-	-	-	-	-	-	-	-	-	-	-	-
Grey Butcherbird	-3.87	-	-	-	-	-	-	-	-	-	-	-	-	-	-	-	-	-	-1.69
Grey Shrike-thrush	-2.29	-	-	-0.61	-	-	-	-	-	-	-	-	-	-	-	-	-	-	-
Grey Whistler	-	-	-	-	-	-	-	-	-	-	-	-	-	-	-	-	-	-	-
Grey-crowned Babbler	-1.73	-	-	-	-	-	-	-	-0.31	-	-	-	-	-	-	-	-	-	-
Grey-fronted Honeyeater	-	-	-	-	-	-	-	-	-	-	-	-	-	-	-	-	-	-	-
Helmeted Friarbird	-2.5	-	-	-	-	0.57	-	-	-	-	-	-	-	-	-	-	-	-2.66	-
Hooded Parrot	-	-	-	-	-	-	-	-	-	-	-	-	-	-	-	-	-	-	-
Hooded Robin	-	-	-	-	-	-	-	-	-	-	-	-	-	-	-	-	-	-	-
Horsfield's Bronze-Cuckoo	-	-	-	-	-	-	-	-	-	-	-	-	-	-	-	-	-	-	-
Jacky Winter	-	-	-	-	-	-	-	-	-	-	-	-	-	-	-	-	-	-	-
Large-tailed Nightjar	-	-	-	-	-	-	-	-	-	-	-	-	-	-	-	-	-	-	-

Species	Int	Clay	Clay^2	Veg. cover	Veg. cover^2	Elev.	Elev.^{2}	Dist. to water.	Terrain rugg.	Max. temp.	Max. temp.^{2}	Ann. rain	Ann. rain^2	Fire freq.	Fire freq.^{2}	Time since fire	Time since fire^2	Fire extent	Fire patch.
Leaden Flycatcher	-0.65	-	-	-	-	-0.94	-0.29	-	-	-	-	-	-	-	-	-0.41	-	-	-
Lemon-bellied Flycatcher	-2.65	-	-	-	-	-	-	-	-	-	-	-	-	-	-	-	-	-0.31	-
Little Bronze-Cuckoo	-2.85	-	-	-	-	-	-	-	-	-	-	-	-	-0.81	-	-	-	-	-
Little Button-quail	-	-	-	-	-	-	-	-	-	-	-	-	-	-	-	-	-	-	-
Little Corella	-4.2	-	-	-	-	-	-	-	-	-	-	-	-	-	-	-	-	-2.04	-
Little Eagle	-	-	-	-	-	-	-	-	-	-	-	-	-	-	-	-	-	-	-
Little Friarbird	-1.09	-	-	-	-	-0.45	-	-	-	0.43	0.35	-	-	-	-	-	-	-	-
Little Pied Cormorant	-	-	-	-	-	-	-	-	-	-	-	-	-	-	-	-	-	-	-
Little Shrike-thrush	-	-	-	-	-	-	-	-	-	-	-	-	-	-	-	-	-	-	-
Little Woodswallow	-1.46	-	-	-	-	-	-	-	-	-	-	0.57	-	-	-	-	-	-	0.38
Long-tailed Finch	-1.9	-	-	-	-	-	-	-	-	-	-	-	-	1.3	-	-	-	-	-
Magpie-lark	-2.45	-	-	-	-	-	-	-	-	-	-	-	-	0.47	-	-	-	-	-
Masked Finch	-2.91	-	-	-	-	-	-	-	-	-0.92	-	-	-	-	-	-	-	-	-
Masked Owl	-	-	-	-	-	-	-	-	-	-	-	-	-	-	-	-	-	-	-
Masked Woodswallow	-1.27	-	-	-	-	0.89	-	-	-	-	-	-	-	-	-	-	-	-	-
Mistletoebird	0.66	-	-	0.43	-	-	-	-	-	0.66	-	-	-	-0.5	-	-0.51	-	-	-
Nankeen Night Heron	-	-	-	-	-	-	-	-	-	-	-	-	-	-	-	-	-	-	-
Northern Fantail	-0.35	-0.41	-	0.36	-	-0.51	-0.57	-	-	-	-	-	-	-	-	-	-	-	-
Northern Rosella	-1.66	-	-	-	-	0.43	-	0.23	-	-	-	-	-	-	-	-	-	-	-
Olive-backed Oriole	-1.71	-	-	-	-	-	-	-	-	-	-	-	-	-0.31	-	-	-	-	-
Orange-footed Scrubfowl	-	-	-	-	-	-	-	-	-	-	-	-	-	-	-	-	-	-	-
Oriental Cuckoo	-	-	-	-	-	-	-	-	-	-	-	-	-	-	-	-	-	-	-
Pallid Cuckoo	-	-	-	-	-	-	-	-	-	-	-	-	-	-	-	-	-	-	-
Partridge Pigeon	-3.31	-	-	0.49	-	-	-	-	-	-	-	-	-	-	-	-	-	-	-
Peaceful Dove	1.59	0.47	-	-	-	-1.63	-	-	0.54	-2.18	-	0.07	0.45	-	-	1.02	-0.7	-	-
Pheasant Coucal	-1.08	-	-	-	-	-	-	-	-0.23	-	-	-	-	-	-	-	-	-	-
Pied Butcherbird	-0.74	-	-	-0.39	-	0.67	-	-	-	-	-	-	-	0.96	-0.5	-	-	-	-
Pied Imperial-Pigeon	-4.29	-	-	-	-	-	-	-1.29	-	-	-	-	-	-	-	-	-	-	-
Radjah Shelduck	-	-	-	-	-	-	-	-	-	-	-	-	-	-	-	-	-	-	-
Rainbow Bee-eater	-1.24	-	-	-	-	0.81	0.72	0.75	-	5.41	1.07	3.75	-	-	-	-	-	-	-0.67
Rainbow Lorikeet	0.71	-	-	-	-	-	-	-0.25	-	-0.22	-0.97	-	-	-	-	-	-	-	-

Species	Int	Clay	Clay^2	Veg. cover	Veg. cover^2	Elev.	Elev.^{2}	Dist. to water.	Terrain rugg.	Max. temp.	Max. temp.^{2}	Ann. rain	Ann. rain^2	Fire freq.	Fire freq.^{2}	Time since fire	Time since fire^2	Fire extent	Fire patch.
Rainbow Pitta	-	-	-	-	-	-	-	-	-	-	-	-	-	-	-	-	-	-	-
Red Goshawk	-	-	-	-	-	-	-	-	-	-	-	-	-	-	-	-	-	-	-
Red-backed Fairy-wren	-2.24	-	-	-	-	-	-	-0.88	-	-	-	-	-	0.51	-	-	-	-	-
Red-backed Kingfisher	-2.61	-	-	-	-	-	-	-	-0.2	-	-	-	-	-	-	-	-	-	-
Red-browed Pardalote	-	-	-	-	-	-	-	-	-	-	-	-	-	-	-	-	-	-	-
Red-tailed Black Cockatoo	-	-	-	-	-	-	-	-	-	-	-	-	-	-	-	-	-	-	-
Red-winged Parrot	-	-	-	-	-	-	-	-	-	-	-	-	-	-	-	-	-	-	-
Restless Flycatcher	-2.53	-	-	-	-	-	-	-	-	-0.57	-	-	-	-	-	-	-	-	-
Rose-crowned Fruit-dove	-	-	-	-	-	-	-	-	-	-	-	-	-	-	-	-	-	-	-
Rufous Owl	-	-	-	-	-	-	-	-	-	-	-	-	-	-	-	-	-	-	-
Rufous Songlark	-4.8	-	-	-	-	-	-	-	-	1.52	-	-	-	-	-	-	-	-	-
Rufous Whistler	-1.86	-	-	-0.47	-	-	-	-	-0.39	2.06	0.69	1.4	-	-	-	-	-	1.43	-
Rufous-banded Honeyeater	-3.74	-	-	-	-	-	-	-1.11	-	-	-	-	-	-	-	-	-	-	-
Rufous-throated Honeyeater	-4	-	-	-	-	-	-	-	-	-	-	-	-	-	-	-	-	-	-
Sacred Kingfisher	-2.59	-	-	-	-	-0.41	-	-	-	-	-	-	-	-	-	-	-	-	-
Sandstone Shrike-thrush	-2.5	-	-	-	-	0.6	-	-	-	-	-	-	-	-	-	-	-	-	-
Shining Flycatcher	-3.57	-	-	-	-	-	-	-	-	-	-	-	-	-	-	0.62	-	-	-
Silver-crowned Friarbird	0.38	0.23	-	-	-	-	-	-	-	-0.8	-0.4	-0.12	0.32	-0.25	-0.26	-	-	-	-
Southern Boobook	0.87	-	-	-	-	1.22	-	2.27	-	-	-	-	-	-	-	-	-	-	-
Spangled Drongo	-1.46	-	-	-	-	-	-	-	-	1.02	-	-	-	-0.88	-	-	-	-	-
Spinifex Pigeon	-	-	-	-	-	-	-	-	-	-	-	-	-	-	-	-	-	-	-
Spotted Harrier	-	-	-	-	-	-	-	-	-	-	-	-	-	-	-	-	-	-	-
Spotted Nightjar	-3.14	-	-	-0.48	-	-	-	-	-	-	-	-	-	-	-	-	-	-	-
Straw-necked ibis	-3.24	-	-	-	-	-	-	-	-	-	-	-	-	-	-	-	-	-0.48	-
Striated Pardalote	0.47	-	-	-	-	-	-	-	-	-	-	0.29	-0.38	-	-	-0.03	-0.56	0.33	-0.54
Sulphur-crested Cockatoo	-1.13	-	-	-	-	-1.59	0.97	-	-	-	-	-	-	-	-	-0.54	-	0.51	-
Tawny Frogmouth	0.25	-	-	-	-	-	-	-	-	-	-	-	-	-	-	-	-	1.06	-
Torresian Crow	-1.29	-	-	-	-	1.62	1.66	-	-0.91	1.22	-	-	-	-	-	-	-	-	-
Tree Martin	-	-	-	-	-	-	-	-	-	-	-	-	-	-	-	-	-	-	-
Varied Lorikeet	-2.41	-	-	-	-	-	-	-	-	-	-	-	-	-	-	-	-	-	-0.79

Species	Int	Clay	Clay^2	Veg. cover	Veg. cover^2	Elev.	Elev.^{2}	Dist. to water.	Terrain rugg.	Max. temp.	Max. temp.^{2}	Ann. rain	Ann. rain^2	Fire freq.	Fire freq.^{2}	Time since fire	Time since fire^2	Fire extent	Fire patch.
Varied Sittella	-	-	-	-	-	-	-	-	-	-	-	-	-	-	-	-	-	-	-
Varied Triller	-	-	-	-	-	-	-	-	-	-	-	-	-	-	-	-	-	-	-
Variegated Fairy-wren	-3.55	-	-	-	-	-	-	-	-	-	-	-	-	-	-	-	-	-	1.02
Wedge-tailed Eagle	-	-	-	-	-	-	-	-	-	-	-	-	-	-	-	-	-	-	-
Weebill	-0.28	-	-	-	-	-0.56	-	-	-	-0.39	-	-	-	0.66	-	-0.4	-	-	-
Whistling Kite	-3.25	-	-	-	-	-1.61	1.22	-	-	-	-	-	-	-	-	0.54	-	-	-
White-bellied Cuckoo-shrike	0.82	-	-	-	-	-2.19	-	-	-	-2.02	-	-1.56	-0.42	0.71	-0.58	-	-	-	-0.68
White-bellied Sea-eagle	-	-	-	-	-	-	-	-	-	-	-	-	-	-	-	-	-	-	-
White-breasted Woodswallow	-3.95	-	-	0.84	-	-	-	-	-	-	-	-	-	-	-	-	-	-	-
White-faced Heron	-	-	-	-	-	-	-	-	-	-	-	-	-	-	-	-	-	-	-
White-gaped Honeyeater	-1.75	-	-	-	-	-0.67	-	-	-0.32	-	-	-	-	-	-	-	-	-	-
White-lined honeyeater	-5.16	-	-	-	-	2.02	-	-	-	1.59	-	-	-	-	-	-	-	-5.27	-
White-throated Gerygone	-	-	-	-	-	-	-	-	-	-	-	-	-	-	-	-	-	-	-
White-throated Grasswren	-	-	-	-	-	-	-	-	-	-	-	-	-	-	-	-	-	-	-
White-throated Honeyeater	0.59	-	-	-	-	-1.04	-	-	-	-1.26	-0.59	-	-	0.81	-	-	-	-	-0.36
White-winged Triller	-2.12	-	-	-	-	-	-	-	-0.59	-	-	-	-	-	-	-	-	-	-
Willie Wagtail	-	-	-	-	-	-	-	-	-	-	-	-	-	-	-	-	-	-	-
Yellow Oriole	-2.5	-	-	-	-	-1.04	-	-	-	0.45	-	-	-	-	-	-	-	-	-
Yellow-throated Miner	-2.64	-	-	-	-	-	-	-	-	-0.41	-	-	-	-	-	-	-	-	-
Yellow-tinted Honeyeater	-	-	-	-	-	-	-	-	-	-	-	-	-	-	-	-	-	-	-
