# Supplementary material for: Occupancy and detectability modelling of vertebrates in northern Australia using multiple sampling methods
Source: PLoS One. 2018 Sep 24;13(9):e0203304. doi: 10.1371/journal.pone.0203304 (PMC6152866; doi:10.1371/journal.pone.0203304)
Supplement: S6 Table — Occupancy and detectability estimates (over one day/night) for 83 birds modelled using diurnal active searches and spotlighting averaged across monitoring sites. (PDF) [file pone.0203304.s012.pdf]

| Species | Ψ mean | Ψ SE | ρ (active searchers Mean) | ρ (active searchers SE) | ρ (spotlighting Mean) | ρ (spotlighting SE) | SSE | Pearson’s | Freeman Tukey |
|---|---|---|---|---|---|---|---|---|---|
| Arafura Fantail | 0.02 | 0.01 | 0.47 | 0.17 | | | 0.43 | 0.31 | 0.44 |
| Australian Owlet-nightjar | 0.42 | 0.19 | 0.02 | 0.01 | 0.07 | 0.03 | 0.52 | 0.14 | 0.51 |
| Banded Fruit-dove | 0.12 | 0.11 | 0.14 | 0.12 | | | 0.47 | 0.42 | 0.44 |
| Banded Honeyeater | 0.07 | 0.03 | 0.39 | 0.10 | | | 0.51 | 0.61 | 0.47 |
| Barking Owl | 0.51 | 0.36 | | | 0.03 | 0.02 | 0.43 | 0.13 | 0.42 |
| Bar-shouldered Dove | 0.48 | 0.08 | 0.54 | 0.03 | | | 0.65 | 0.95 | 0.6 |
| Black-faced Cuckoo-shrike | 0.17 | 0.06 | 0.32 | 0.06 | | | 0.44 | 0.76 | 0.47 |
| Black-shouldered Kite | 0.02 | 0.01 | 0.47 | 0.17 | | | 0.42 | 0.11 | 0.41 |
| Black-tailed Treecreeper | 0.05 | 0.02 | 0.51 | 0.11 | | | 0.50 | 0.73 | 0.49 |
| Blue-faced Honeyeater | 0.26 | 0.08 | 0.25 | 0.06 | | | 0.46 | 0.77 | 0.45 |
| Blue-winged Kookaburra | 0.34 | 0.1 | 0.24 | 0.05 | | | 0.53 | 0.47 | 0.5 |
| Brown Falcon | 0.2 | 0.13 | 0.11 | 0.07 | | | 0.47 | 0.54 | 0.48 |
| Brown Goshawk | 0.45 | 0.43 | 0.06 | 0.05 | | | 0.48 | 0.78 | 0.47 |
| Brown Honeyeater | 0.37 | 0.06 | 0.61 | 0.05 | | | 0.45 | 0.58 | 0.44 |
| Brown Quail | 0.13 | 0.07 | 0.17 | 0.09 | | | 0.46 | 0.57 | 0.46 |
| Chestnut-backed Button-quail | 0.06 | 0.05 | 0.16 | 0.11 | | | 0.39 | 0.53 | 0.41 |
| Chestnut-quilled Rock-Pigeon | 0.11 | 0.04 | 0.40 | 0.09 | | | 0.43 | 0.14 | 0.44 |
| Cicadabird | 0.14 | 0.14 | 0.10 | 0.09 | | | 0.46 | 0.71 | 0.44 |
| Collared Sparrowhawk | 0.31 | 0.8 | 0.01 | 0.03 | | | 0.44 | 0.36 | 0.43 |
| Common Bronzewing | 0.05 | 0.03 | 0.23 | 0.13 | | | 0.45 | 0.46 | 0.46 |
| Crimson Finch | 0.08 | 0.03 | 0.35 | 0.10 | | | 0.49 | 0.49 | 0.48 |
| Diamond Dove | 0.11 | 0.04 | 0.31 | 0.07 | | | 0.30 | 0.37 | 0.42 |
| Dollarbird | 0.04 | 0.03 | 0.28 | 0.15 | | | 0.42 | 0.18 | 0.42 |
| Double-barred Finch | 0.06 | 0.05 | 0.16 | 0.14 | | | 0.51 | 0.42 | 0.45 |
| Forest Kingfisher | 0.13 | 0.04 | 0.44 | 0.07 | | | 0.43 | 0.47 | 0.45 |
| Galah | 0.11 | 0.06 | 0.20 | 0.09 | | | 0.45 | 0.48 | 0.46 |
| Golden-headed Cisticola | 0.06 | 0.02 | 0.45 | 0.11 | | | 0.51 | 0.11 | 0.49 |
| Grey Butcherbird | 0.05 | 0.02 | 0.53 | 0.11 | | | 0.50 | 0.90 | 0.46 |
| Grey Shrike-thrush | 0.10 | 0.04 | 0.30 | 0.09 | | | 0.48 | 0.26 | 0.47 |

| Species | Ψ mean | Ψ SE | ρ (active searchers Mean) | ρ (active searchers SE) | ρ (spotlighting Mean) | ρ (spotlighting SE) | SSE | Pearson's | Freeman Tukey |
|---|---|---|---|---|---|---|---|---|---|
| Grey-crowned Babbler | 0.15 | 0.06 | 0.21 | 0.08 | | | 0.50 | 0.83 | 0.48 |
| Helmeted Friarbird | 0.18 | 0.04 | 0.66 | 0.07 | | | 0.49 | 0.57 | 0.44 |
| Leaden Flycatcher | 0.32 | 0.09 | 0.26 | 0.06 | | | 0.48 | 0.58 | 0.47 |
| Lemon-bellied Flycatcher | 0.07 | 0.02 | 0.63 | 0.08 | | | 0.49 | 0.55 | 0.47 |
| Little Bronze-Cuckoo | 0.07 | 0.05 | 0.20 | 0.12 | | | 0.46 | 0.66 | 0.45 |
| Little Corella | 0.03 | 0.02 | 0.32 | 0.17 | | | 0.48 | 0.59 | 0.46 |
| Little Friarbird | 0.34 | 0.09 | 0.29 | 0.06 | | | 0.55 | 0.30 | 0.54 |
| Little Woodswallow | 0.20 | 0.08 | 0.20 | 0.07 | | | 0.45 | 0.83 | 0.45 |
| Long-tailed Finch | 0.19 | 0.14 | 0.09 | 0.07 | | | 0.48 | 0.52 | 0.46 |
| Magpie-lark | 0.09 | 0.03 | 0.46 | 0.08 | | | 0.38 | 0.40 | 0.43 |
| Masked Finch | 0.07 | 0.08 | 0.09 | 0.09 | | | 0.53 | 0.15 | 0.49 |
| Masked Woodswallow | 0.25 | 0.53 | 0.02 | 0.04 | | | 0.41 | 0.24 | 0.40 |
| Mistletoebird | 0.64 | 0.08 | 0.51 | 0.04 | | | 0.48 | 0.81 | 0.49 |
| Northern Fantail | 0.31 | 0.07 | 0.42 | 0.05 | | | 0.52 | 0.22 | 0.50 |
| Northern Rosella | 0.17 | 0.06 | 0.30 | 0.07 | | | 0.49 | 0.27 | 0.47 |
| Olive-backed Oriole | 0.16 | 0.10 | 0.13 | 0.08 | | | 0.52 | 0.81 | 0.49 |
| Partridge Pigeon | 0.04 | 0.03 | 0.29 | 0.15 | | | 0.52 | 0.72 | 0.45 |
| Peaceful Dove | 0.68 | 0.09 | 0.52 | 0.04 | | | 0.21 | 0.20 | 0.17 |
| Pheasant Coucal | 0.26 | 0.24 | 0.08 | 0.07 | | | 0.50 | 0.62 | 0.46 |
| Pied Butcherbird | 0.28 | 0.07 | 0.44 | 0.04 | | | 0.57 | 0.21 | 0.55 |
| Pied Imperial-Pigeon | 0.02 | 0.01 | 0.55 | 0.15 | | | 0.48 | 0.55 | 0.46 |
| Rainbow Bee-eater | 0.54 | 0.08 | 0.42 | 0.03 | | | 0.49 | 0.51 | 0.48 |
| Rainbow Lorikeet | 0.49 | 0.07 | 0.45 | 0.05 | | | 0.47 | 0.58 | 0.41 |
| Red-backed Fairy-wren | 0.13 | 0.04 | 0.38 | 0.07 | | | 0.58 | 0.09 | 0.51 |
| Red-backed Kingfisher | 0.07 | 0.07 | 0.11 | 0.10 | | | 0.39 | 0.16 | 0.45 |
| Restless Flycatcher | 0.08 | 0.03 | 0.33 | 0.09 | | | 0.43 | 0.12 | 0.45 |
| Rufous Songlark | 0.02 | 0.01 | 0.44 | 0.15 | | | 0.36 | 0.23 | 0.39 |
| Rufous Whistler | 0.32 | 0.08 | 0.46 | 0.04 | | | 0.42 | 0.68 | 0.43 |
| Rufous-banded Honeyeater | 0.04 | 0.02 | 0.74 | 0.10 | | | 0.54 | 0.75 | 0.51 |

| Species | Ψ mean | Ψ SE | ρ (active searchers Mean) | ρ (active searchers SE) | ρ (spotlighting Mean) | ρ (spotlighting SE) | SSE | Pearson's | Freeman Tukey |
|---|---|---|---|---|---|---|---|---|---|
| Rufous-throated Honeyeater | 0.04 | 0.03 | 0.24 | 0.14 | | | 0.48 | 0.63 | 0.47 |
| Sacred Kingfisher | 0.07 | 0.04 | 0.24 | 0.11 | | | 0.51 | 0.23 | 0.48 |
| Sandstone Shrike-thrush | 0.09 | 0.03 | 0.52 | 0.08 | | | 0.57 | 0.71 | 0.49 |
| Shining Flycatcher | 0.03 | 0.02 | 0.54 | 0.13 | | | 0.48 | 0.88 | 0.48 |
| Silver-crowned Friarbird | 0.51 | 0.09 | 0.53 | 0.03 | | | 0.55 | 0.80 | 0.51 |
| Southern Boobook | 0.56 | 0.12 | | | 0.14 | 0.03 | 0.48 | 0.36 | 0.47 |
| Spangled Drongo | 0.27 | 0.07 | 0.31 | 0.06 | | | 0.43 | 0.31 | 0.42 |
| Spotted Nightjar | 0.05 | 0.03 | | | 0.44 | 0.21 | 0.40 | 0.21 | 0.43 |
| Straw-necked ibis | 0.04 | 0.03 | 0.28 | 0.15 | | | 0.49 | 0.60 | 0.44 |
| Striated Pardalote | 0.45 | 0.08 | 0.55 | 0.03 | | | 0.40 | 0.70 | 0.43 |
| Sulphur-crested Cockatoo | 0.47 | 0.13 | 0.23 | 0.05 | | | 0.52 | 0.69 | 0.48 |
| Tawny Frogmouth | 0.53 | 0.27 | 0.02 | 0.01 | 0.09 | 0.04 | 0.49 | 0.09 | 0.46 |
| Torresian Crow | 0.59 | 0.12 | 0.24 | 0.06 | | | 0.51 | 0.25 | 0.46 |
| Varied Lorikeet | 0.10 | 0.04 | 0.28 | 0.09 | | | 0.54 | 0.64 | 0.48 |
| Variegated Fairy-wren | 0.05 | 0.02 | 0.53 | 0.12 | | | 0.49 | 0.85 | 0.48 |
| Weebill | 0.45 | 0.07 | 0.55 | 0.03 | | | 0.36 | 0.57 | 0.38 |
| Whistling Kite | 0.22 | 0.06 | 0.34 | 0.06 | | | 0.69 | 0.46 | 0.65 |
| White-bellied Cuckoo-shrike | 0.71 | 0.08 | 0.29 | 0.03 | | | 0.46 | 0.42 | 0.44 |
| White-breasted Woodswallow | 0.03 | 0.03 | 0.20 | 0.17 | | | 0.39 | 0.01 | 0.44 |
| White-gaped Honeyeater | 0.17 | 0.04 | 0.53 | 0.08 | | | 0.46 | 0.07 | 0.39 |
| White-lined honeyeater | 0.15 | 0.03 | 0.67 | 0.06 | | | 0.25 | 0.66 | 0.33 |
| White-throated Honeyeater | 0.52 | 0.07 | 0.63 | 0.04 | | | 0.50 | 0.90 | 0.54 |
| White-winged Triller | 0.12 | 0.05 | 0.24 | 0.08 | | | 0.40 | 0.06 | 0.42 |
| Yellow Oriole | 0.13 | 0.04 | 0.43 | 0.07 | | | 0.49 | 0.11 | 0.48 |
| Yellow-throated Miner | 0.07 | 0.03 | 0.45 | 0.09 | | | 0.47 | 0.72 | 0.44 |
