# Supplementary material for: Occupancy and detectability modelling of vertebrates in northern Australia using multiple sampling methods
Source: PLoS One. 2018 Sep 24;13(9):e0203304. doi: 10.1371/journal.pone.0203304 (PMC6152866; doi:10.1371/journal.pone.0203304)
Supplement: S7 Table — Occupancy and detectability (over one day/night) estimates for 33 reptiles modelled using pitfall trapping and/or spotlighting data averaged across monitoring sites. (PDF) [file pone.0203304.s013.pdf]

Species	Ψ mean	Ψ SE	ρ (pit trapping Mean)	ρ (pit trapping SE)	ρ (spotlighting Mean)	ρ (spotlighting SE)	SSE	Pearson's	Freeman Tukey
Carlia amax	0.49	0.07	0.47	0.05			0.75	0.25	0.66
Carlia gracilis	0.15	0.05	0.43	0.07			0.48	0.82	0.47
Carlia munda	0.34	0.07	0.46	0.05			0.59	0.98	0.53
Carlia rufilatus	0.02	0.01	0.38	0.18			0.45	0.48	0.44
Ctenophorus caudicinctus	0.03	0.01	0.54	0.18			0.75	0.75	0.60
Ctenotus coggeri	0.34	0.12	0.36	0.09			0.53	0.70	0.46
Ctenotus decaneurus	0.05	0.03	0.22	0.10			0.39	0.07	0.42
Ctenotus essingtonii	0.21	0.05	0.37	0.05			0.52	0.22	0.49
Ctenotus inornatus	0.07	0.03	0.31	0.08			0.36	0.63	0.42
Ctenotus pantherinus	0.01	0.01	0.60	0.19			0.39	0.45	0.41
Ctenotus piankai	0.02	0.01	0.69	0.11			0.35	0.24	0.37
Ctenotus robustus	0.03	0.02	0.33	0.12			0.35	0.12	0.40
Ctenotus spaldingi	0.05	0.03	0.25	0.10			0.45	0.51	0.48
Ctenotus storri	0.01	0.01	0.57	0.21			0.53	0.57	0.49
Ctenotus vertebralis	0.39	0.11	0.57	0.07			0.61	0.94	0.52
Eremiscincus isolepis	0.34	0.12	0.07	0.03			0.49	0.53	0.47
Gehyra australis	0.33	0.12	0.08	0.03	0.13	0.05	0.52	0.87	0.51
Gehyra nana	0.14	0.06	0.15	0.06	0.10	0.05	0.49	0.83	0.49
Gehyra pamela	0.19	0.06			0.45	0.12	0.49	0.75	0.48
Glaphyromorphus darwiniensis	0.41	0.36	0.02	0.02			0.45	0.19	0.45
Heteronotia binoei	0.47	0.09	0.27	0.05	0.28	0.09	0.62	0.46	0.55
Heteronotia planiceps	0.29	0.42	0.02	0.03			0.46	0.42	0.45
Lerista orientalis	0.03	0.02	0.23	0.18			0.51	0.65	0.45
Menetia greyii	0.04	0.03	0.22	0.13			0.49	0.22	0.47
Menetia maini	0.10	0.04	0.24	0.08			0.49	0.66	0.49
Morethia ruficauda	0.08	0.03	0.32	0.10			0.60	0.66	0.53
Morethia storii	0.11	0.01	0.14	0.07			0.42	0.06	0.43
Notoscincus ornatus	0.10	0.03	0.30	0.08			0.45	0.53	0.47
Oedura gemmata	0.21	0.18			0.18	0.16	0.41	0.48	0.48
Oedura marmorata	0.03	0.01			0.57	0.22	0.47	0.52	0.48
Pseudothecadactylus lindneri	0.14	0.10	0.22	0.16			0.57	0.79	0.49
Varanus baritji	0.22	0.67	0.04	0.13			0.50	0.75	0.45
Varanus primordius	0.17	0.19	0.04	0.06			0.57	0.63	0.51
