# Supplementary material for: Occupancy and detectability modelling of vertebrates in northern Australia using multiple sampling methods
Source: PLoS One. 2018 Sep 24;13(9):e0203304. doi: 10.1371/journal.pone.0203304 (PMC6152866; doi:10.1371/journal.pone.0203304)
Supplement: S8 Table — Occupancy and detectability for 20 mammals modelled using live trapping and spotlighting averaged across 326 sites, and camera trapping averaged across 168 sites in northern Australia. Detectability estimates for live trapping and spotlighting is over the period of a day/night, and one week for camera trapping. * denotes introduced species. (PDF) [file pone.0203304.s014.pdf]

Species	Ψ mean	Ψ SE	ρ (Spotlight Mean)	ρ (Spotlight SE)	ρ (Pit, Mean)	ρ (Pit, SE)	ρ (Elliot, Mean)	ρ (Elliot, SE	ρ (Cage, Mean)	ρ (Cage, SE	ρ (Camera, Mean)	ρ (Camera, SE	SSE	Pearson’s	Freeman Tukey
Agile Wallaby	0.30	0.06									0.49	0.06	0.65	0.66	0.53
Antilopine Wallaroo	0.14	0.04									0.27	0.05	0.53	0.21	0.52
Arnhem Land Rock Rat	0.13	0.03			0.04	0.03	0.57	0.08	0.02	0.02	0.36	0.15	0.90	0.70	0.69
Black Rat*	0.08	0.02							0.06	0.03	0.49	0.06	0.34	0.09	0.41
Black Wallaroo	0.13	0.05									0.53	0.08	0.39	0.70	0.39
Black-footed Tree-rat	0.18	0.04							0.11	0.02	0.68	0.03	0.11	0.35	0.44
Common Brushtail Possum	0.12	0.04	0.05	0.04							0.47	0.04	0.33	0.14	0.35
Common Planigale	0.15	0.12			0.08	0.06							0.46	0.17	0.46
Common Rock Rat	0.16	0.04			0.01	0.01	0.34	0.05	0.04	0.02	0.34	0.06	0.76	0.19	0.65
Common Wallaroo	0.29	0.06									0.41	0.05	0.60	0.40	0.45
Delicate Mouse	0.12	0.11			0.05	0.05	0.01	0.01			0.02	0.02	0.49	0.20	0.49
Grassland Melomys	0.13	0.03			0.01	0.01	0.18	0.05	0.08	0.03	0.18	0.06	0.76	0.77	0.64
Northern Brown Bandicoot	0.18	0.04							0.23	0.03	0.85	0.02	0.70	0.73	0.68
Northern Quoll	0.02	0.02							0.08	0.07	0.12	0.08	0.36	0.09	0.41
Pale Field Rat	0.03	0.02					0.25	0.13	0.03	0.04	0.05	0.05	0.60	0.52	0.53
Red-cheeked Dunnart	0.04	0.02			0.09	0.06	0.09	0.06			0.05	0.04	0.44	0.47	0.46
Sandstone Antechinus	0.08	0.03	0.03	0.03			0.19	0.09			0.04	0.04	0.62	0.68	0.56
Short-beaked Echidna	0.13	0.04									0.19	0.06	0.51	0.81	0.51
Short-eared rock Wallaby	0.02	0.01									0.94	0.06	0.34	0.37	0.33
Sugar Glider	0.04	0.01	0.67	0.16									0.50	0.53	0.47
