# Supplementary material for: Occupancy and detectability modelling of vertebrates in northern Australia using multiple sampling methods
Source: PLoS One. 2018 Sep 24;13(9):e0203304. doi: 10.1371/journal.pone.0203304 (PMC6152866; doi:10.1371/journal.pone.0203304)
Supplement: S9 Table — Note, species containing only dashes were recorded during surveys but were unable to be modelled. (PDF) [file pone.0203304.s015.pdf]

Species	Intercept	Terrain ruggedness	Fire frequency	Time since fire	Sampling Method	Model number	Comment	Occupied Sites
Carlia amax	-0.09	0.48	-	0.22	-	4		119
Carlia gracilis	-0.27	-	-	-	-	7		31
Carlia munda	-0.18	-	-0.2	-	-	1		83
Carlia rufilatus	-0.5	-	-	-	-	4		4
Carlia triacantha	-	-	-	-	-		No suitable models	7
Chlamydosaurus kingii	-	-	-	-	-		Failed GOF tests	3
Cryptoblepharus sp.	-	-	-	-	-		No suitable models	89
Ctenophorus caudicinctus	0.16	-	-	-	-	2		7
Ctenotus arnhemensis	-	-	-	-	-		Could not fit models	4
Ctenotus borealis	-	-	-	-	-		Could not fit models	3
Ctenotus coggeri	-0.6	-	-	-	-	1		29
Ctenotus decaneurus	-1.28	-	-	-	-	7		8
Ctenotus essingtonii	-0.51	-	-	-	-	3		46
Ctenotus inornatus	-0.8	-	-	-	-	1		15
Ctenotus pantherinus	0.41	-	-	-	-	3		2
Ctenotus piankai	0.79	-	-	-	-	4		5
Ctenotus quirinus	-	-	-	-	-		Could not fit models	1
Ctenotus robustus	-0.69	-	-	-	-	2		6
Ctenotus saxatilis	-	-	-	-	-		No suitable models	2
Ctenotus spaldingi	-1.1	-	-	-	-	3		9
Ctenotus storri	0.28	-	-	-	-	3		3
Ctenotus vertebralis	0.61	1.85	-	-	-	4		41
Delma borea	-	-	-	-	-		Could not fit models	9
Delma tincta	-	-	-	-	-		Could not fit models	2
Diplodactylus bilineata	-	-	-	-	-		Could not fit models	1
Diplodactylus conspicillatus	-	-	-	-	-		Could not fit models	1
Diporiphora albilabris	-	-	-	-	-		Could not fit models	2
Diporiphora bennettii	-	-	-	-	-		Could not fit models	2

Species	Intercept	Terrain ruggedness	Fire frequency	Time since fire	Sampling Method	Model number	Comment	Occupied Sites
Diporiphora bilineata	-	-	-	-	-		Could not fit models	45
Diporiphora magna	-	-	-	-	-		No suitable models	2
Eremiscincus isolepis	-2.61	-	-	-	-	1		19
Gehyra australis	-1.93	-	-	-	+	3		35
Gehyra nana	-2.15	-	-	-	+	5		20
Gehyra pamela	-0.19	-	-	-	+	7		16
Glaphyromorphus darwiniensis	-3.99	-	-	-	-	10		5
Glaphyromorphus douglasi	-	-	-	-	-		Could not fit models	1
Hemidactylus frenatus	-	-	-	-	-		Could not fit models	1
Heteronotia binoei	-0.97	-0.39	-	0.38	+	2		81
Heteronotia planiceps	-3.85	-	-	-	-	7		5
Lerista karlschmidti	-	-	-	-	-		Failed GOF tests	2
Lerista orientalis	-1.22	-	-	-	-	4		4
Lialis burtonis	-	-	-	-	-		Could not fit models	5
Lophognathus gilberti	-	-	-	-	-		Failed GOF tests	4
Lophognathus temporalis	-	-	-	-	-		Could not fit models	2
Lucasium stenodactylum	-	-	-	-	-		No suitable models	2
Menetia alanae	-	-	-	-	-		Failed GOF tests	3
Menetia concinna	-	-	-	-	-		Could not fit models	1
Menetia greyii	-1.3	-	-	-	-	7		6
Menetia maini	-1.17	-	-	-	-	1		17
Morethia ruficauda	-0.71	-	-	-	-	0	Null model	16
Morethia storri	-1.98	-	-	-	-	0	Null model	13
Nephrurus sheai	-	-	-	-	-		Could not fit models	3
Notoscincus ornatus	-0.83	-	-	-	-	1		19
Oedura gemmata	-1.5	-	-	-	+	0	Null model	6
Oedura marmorata	0.29	-	-	-	+	0	Null model	4

Species	Intercept	Terrain ruggedness	Fire frequency	Time since fire	Sampling Method	Model number	Comment	Occupied Sites
Oedura rhombifer	-	-	-	-	-		Failed GOF tests	9
Proablepharus tenuis	-	-	-	-	-		No suitable models	31
Pseudonaja nuchalis	-	-	-	-	-		Could not fit models	2
Pseudothecadactylus lindneri	-1.26	-	-	-	-	4		10
Ramphotyphlops sp.	-	-	-	-	-		Could not fit models	4
Sphenomorphus sp.	-	-	-	-	-		Could not fit models	1
Strophurus ciliaris	-	-	-	-	-		Failed GOF tests	7
Strophurus taeniatus	-	-	-	-	-		Failed GOF tests	4
Varanus acanthurus	-	-	-	-	-		Could not fit models	1
Varanus baritji	-3.73	-	-	-	-	0	Null model	6
Varanus kingorum	-	-	-	-	-		Could not fit models	1
Varanus primordius	-1.59	-	-	-	-	3		5
Varanus scalaris	-	-	-	-	-		Failed GOF tests	9
Varanus tristis	-	-	-	-	-		Could not fit models	1
