# Supplementary material for: Occupancy and detectability modelling of vertebrates in northern Australia using multiple sampling methods
Source: PLoS One. 2018 Sep 24;13(9):e0203304. doi: 10.1371/journal.pone.0203304 (PMC6152866; doi:10.1371/journal.pone.0203304)
Supplement: S10 Table — Note, species containing only dashes were recorded during surveys but were unable to be modelled. (PDF) [file pone.0203304.s016.pdf]

Species	Intercept	Terrain ruggedness	Fire Frequency	Time since fire	Sampling Method	Model number	Comment	Occupied Sites
Apostlebird							Could not fit models	1
Arafura Fantail	-0.13					0	Null model	4
Australasian Darter							Could not fit models	1
Australasian Figbird							Could not fit models	1
Australian Bustard							Could not fit models	1
Australian Hobby							No suitable models	2
Australian Owlet-nightjar	-2.56				+	4		20
Australian Pelican							Could not fit models	1
Australian White Ibis							Failed GOF tests	2
Azure Kingfisher							Could not fit models	3
Banded Fruit-dove	-1.8					10		11
Banded Honeyeater	-0.47					2		13
Bar-breasted Honeyeater							No suitable models	2
Barking Owl	-3.56					2		6
Bar-shouldered Dove	0.15					1		100
Black Bittern							Could not fit models	1
Black Kite							Could not fit models	3
Black-breasted Buzzard							Could not fit models	1
Black-chinned Honeyeater							Could not fit models	1
Black-faced Cuckoo-shrike	-0.76					1		28
Black-faced Woodswallow							Failed GOF tests	10
Black-shouldered Kite	-0.11					2		4
Black-tailed Treecreeper	0.04					1		11
Blue-faced Honeyeater	-1.12					6		35
Blue-winged Kookaburra	-1.16					1		45
Broad-billed Flycatcher							No suitable models	2
Brown Falcon	-2.11					3		14
Brown Goshawk	-2.74					7		20
Brown Honeyeater	0.44		0.26			2		81
Brown Quail	-1.57					4		14
Brush Cuckoo							Could not fit models	19
Bush Stone-curlew							Could not fit models	6

Species	Intercept	Terrain ruggedness	Fire frequency	Time since fire	Sampling Method	Model number	Comment	Occupied Sites
Cattle Egret							Could not fit models	1
Channel-billed Cuckoo							No suitable models	3
Chestnut-backed Button-quail	-1.69					4		6
Chestnut-breasted Mannikin							Could not fit models	1
Chestnut-quilled Rock-Pigeon	-0.41					11		20
Cicadabird	-2.21					7		9
Collared Sparrowhawk	-4.29					9		3
Common Bronzewing	-1.23					1		6
Crimson Finch	-0.63					1		13
Diamond Dove	-1.62					4		18
Dollarbird	-0.79					1		6
Double-barred Finch	-1.62					5		6
Dusky Grasswren							Could not fit models	1
Dusky Honeyeater							No suitable models	62
Eastern Great Egret							Could not fit models	2
Eastern Koel							Could not fit models	1
Emerald Dove							Could not fit models	2
Forest Kingfisher	-0.24					1		26
Fork-tailed Swift							Could not fit models	1
Galah	-1.4					1		13
Golden-headed Cisticola	-0.21					5		11
Great Bowerbird							No suitable models	47
Green-backed Gerygone							No suitable models	10
Grey Butcherbird	0.11					1		10
Grey Shrike-thrush	-0.85					4		16
Grey Whistler							Could not fit models	1
Grey-crowned Babbler	-1.31					5		19
Grey-fronted Honeyeater							No suitable models	3
Helmeted Friarbird	0.71	0.58				2		37
Hooded Parrot							No suitable models	2
Hooded Robin							Could not fit models	1
Horsfield's Bronze-Cuckoo							Could not fit models	1
Jacky Winter							Could not fit models	5
Large-tailed Nightjar							Could not fit models	1

Species	Intercept	Terrain ruggedness	Fire frequency	Time since fire	Sampling Method	Model number	Comment	Occupied Sites
Little Bronze-Cuckoo	-1.38					2		8
Little Button-quail							Could not fit models	1
Little Corella	-0.74					3		5
Little Eagle							Could not fit models	1
Little Friarbird	-0.96			-0.49		3		48
Little Pied Cormorant							Could not fit models	2
Little Shrike-thrush							Could not fit models	5
Little Woodswallow	-1.38					4		24
Long-tailed Finch	-2.32					3		12
Magpie-lark	-0.18					6		17
Masked Finch	-2.34					3		5
Masked Owl							Failed GOF tests	3
Masked Woodswallow	-3.84					4		4
Mistletoebird	0.06			0.25		1		131
Nankeen Night Heron							Could not fit models	2
Northern Fantail	-0.34					1		59
Northern Rosella	-0.86					6		27
Olive-backed Oriole	-1.88					4		13
Orange-footed Scrubfowl							No suitable models	7
Oriental Cuckoo							Could not fit models	1
Pallid Cuckoo							Could not fit models	1
Partridge Pigeon	-0.89					3		6
Peaceful Dove	0.06		0.4			1		140
Pheasant Coucal	-2.51					4		13
Pied Butcherbird	-0.24					1		55
Pied Imperial-Pigeon	0.2					1		5
Radjah Shelduck							Could not fit models	1
Rainbow Bee-eater	-0.34					1		100
Rainbow Lorikeet	-0.21		-0.37	-0.7		1		92
Rainbow Pitta							No suitable models	5
Red Goshawk							Could not fit models	3
Red-backed Fairy-wren	-0.48					1		24
Red-backed Kingfisher	-2.07					12		5
Red-browed Pardalote							Could not fit models	6

Species	Intercept	Terrain ruggedness	Fire frequency	Time since fire	Sampling Method	Model number	Comment	Occupied Sites
Red-tailed Black Cockatoo							Could not fit models	4
Red-winged Parrot							Could not fit models	73
Restless Flycatcher	-0.73					2		14
Rose-crowned Fruit-dove							Could not fit models	2
Rufous Owl							Could not fit models	1
Rufous Songlark	-0.23					3		4
Rufous Whistler	-0.15					1		61
Rufous-banded Honeyeater	1.04					5		8
Rufous-throated Honeyeater	-1.13					0	Null model	6
Sacred Kingfisher	-1.13					3		10
Sandstone Shrike-thrush	0.07					9		18
Shining Flycatcher	0.15					2		7
Silver-crowned Friarbird	0.13					1		107
Southern Boobook	-1.85					9		33
Spangled Drongo	-0.82					3		41
Spinifex Pigeon							Could not fit models	1
Spotted Harrier							Could not fit models	1
Spotted Nightjar	-0.23					1		7
Straw-necked ibis	-0.93					7		6
Striated Pardalote	0.22					1		95
Sulphur-crested Cockatoo	-1.27		-0.38			13		54
Tawny Frogmouth	-2.36				+	5		27
Torresian Crow	0.82		-1.29	-0.65		1		64
Tree Martin							Could not fit models	1
Varied Lorikeet	-0.94					1		15
Varied Sittella						0	Null model	4
Varied Triller							Failed GOF tests	19
Variegated Fairy-wren	0.13					1		11
Wedge-tailed Eagle							Could not fit models	2
Weebill	0.21					1		96
Whistling Kite	-0.65					1		37
White-bellied Cuckoo-shrike	-0.57	0.29				1		110
White-bellied Sea-eagle							Could not fit models	2
White-breasted Woodswallow	-1.41					2		3

Species	Intercept	Terrain ruggedness	Fire frequency	Time since fire	Sampling Method	Model number	Comment	Occupied Sites
White-breasted Woodswallow	-1.41					2		3
White-faced Heron							Could not fit models	1
White-gaped Honeyeater	0.12		0.47			4		34
White-lined honeyeater	0.72					1		32
White-throated Gerygone							Failed GOF tests	3
White-throated Grasswren							Could not fit models	3
White-throated Honeyeater	0.53	-0.21				1		113
White-winged Triller	-1.14					2		16
Willie Wagtail							No suitable models	46
Yellow Oriole	-0.3					6		24
Yellow-throated Miner	-0.22					4		14
Yellow-tinted Honeyeater							Could not fit models	1
