# Supplementary material for: Occupancy and detectability modelling of vertebrates in northern Australia using multiple sampling methods
Source: PLoS One. 2018 Sep 24;13(9):e0203304. doi: 10.1371/journal.pone.0203304 (PMC6152866; doi:10.1371/journal.pone.0203304)
Supplement: S11 Table — Note, species containing only dashes were recorded during surveys but were unable to be modelled. (PDF) [file pone.0203304.s017.pdf]

Species	Intercept	Terrain ruggedness	Fire frequency	Time since fire	Sampling Method	Model number	Comment	Occupied Sites
Agile Wallaby	-0.13	-0.9	-0.28	-	-	1		47
Antilopine Wallaroo	-1.04	-	-	-	-	2		19
Arnhem Land Rock Rat	-3.14	-	-	-	+	0	Null model	20
Black Rat	-11.54	-	-	-	+	0	Null model	15
Black Wallaroo	0.1	-	-	-	-	3		26
Black-footed Tree-rat	-2.08	-	-	-	-	1		34
Brush-tailed Rabbit-rat	-	-	-	-	-	-	Failed GOF test	18
Central Pebble-mound Mouse	-	-	-	-	-	-	Could not fit models	3
Common Brushtail Possum	-3.76	-	-	-	+	6		31
Common Planigale	-3.29	-	-	-	-	2		11
Common Rock Rat	-5.12	-	-0.37	-	+	5		42
Common Wallaroo	-0.37	-0.49	-	-	-	3		43
Delicate Mouse	-2.96	-	-	-	+	0	Null model	8
Grassland Melomys	-4.61	-	-	-	+	6		27
Northern Brown Bandicoot	-1.22	-	-	-	+	2		47
Northern Nailtail Wallaby	-	-	-	-	-	-	No suitable models	2
Northern Quoll	-10.7	-	-	-	+	0	Null model	3
Pale Field Rat	-1.11	-	-	-	+	0	Null model	7
Red-cheeked Dunnart	-2.28	-	-	-	+	0	Null model	7
Rock Ringtail Possum	-	-	-	-	-	-	Failed GOF test	1
Sandstone Antechinus	-3.51	-	-	-	+	0	Null model	13
Short-beaked Echidna	-10.68	-	-	-	+	0	Null model	14
Short-eared Rock Wallaby	-4.96	-	-	-	-	3		11
Sugar Glider	0.69	-	-	-	-	2		8
Water Rat	-	-	-	-	-	-	No suitable models	4
Western Chestnut Mouse	-	-	-	-	-	-	Failed GOF test	7
